# Chemical Fractionation in Environmental Studies of Potentially Toxic Particulate-Bound Elements in Urban Air: A Critical Review

**DOI:** 10.3390/toxics10030124

**Published:** 2022-03-04

**Authors:** Ryszard Świetlik, Marzena Trojanowska

**Affiliations:** Department of Environmental Protection, Kazimierz Pulaski University of Technology and Humanities in Radom, Chrobrego 27, 26-600 Radom, Poland; m.trojanowska@uthrad.pl

**Keywords:** potentially toxic elements, trace elements, heavy metals, chemical fractionation, sequential extraction, urban air, PM, ecological risk, inhalation health risk

## Abstract

In recent years, studies of heavy metal air pollution have increasingly gone beyond determining total concentrations of individual toxic metals. Chemical fractionation of potentially toxic elements in airborne particles is becoming an important part of these studies. This review covers the articles that have been published over the last three decades. Attention was paid to the issue of atmospheric aerosol sampling, sample pretreatment, sequential extraction schemes and conditions of individual extractions. Geochemical forms of metals occurring in the air in urban areas were considered in detail. Based on the data sets from chemical fractionation of particulate matter samples by three sequential extraction procedures (SEPs)—Fernández Espinosa, BCR and Chester’s—the compilation of the chemical distribution patterns of As, Cd, Co, Cr, Cu, Fe, Mn, Ni, Pb and Zn was prepared. The human health risk posed by these toxic and/or carcinogenic elements via inhalation of atmospheric particles was estimated for two categories of polluted urban areas: the commonly encountered pollution level and the high pollution level.

## 1. Introduction

Among the primary air pollutants (CO, CO_2_, CH_4_, VOCs, NO, N_2_O, NH_3_, H_2_S, SO_2_ and particulate matter) and the secondary air pollutants (NO_2_, HNO_3_, O_3_, H_2_SO_4_, sulfate, nitrate and organic aerosols), atmospheric particulate matter (APM) is of special interest to researchers due to its adverse effect on human health [1]. In urban and industrial areas, APM is a mixture of particles originating from natural sources (seas and oceans, deserts, soil, volcanes, vegetation and wildfires) and from anthropogenic sources (traffic, industrial activity, building and housing). In rural areas, APM additionally originates from biomass burning and farming activity [1]. The composition of APM is complex and it depends on local emission sources. Major urban-APM constituents are organic matter, sulfate, mineral dust, nitrate, ammonium, elemental carbon (including black carbon) and chloride [2]. Particularly important constituents of APM are trace elements, because many of them are toxic and carcinogenic, the so-called potentially toxic elements (PTEs) [3].

The goal of this paper is to review the present state of knowledge on the distribution of chemical forms of potentially toxic elements in airborne particles. Earlier publications focused on the methodology of chemical fractionation [4,5]. The results of operational studies of PTE speciation in urban air have not been the subject of review elaborations so far. Formally, this review covers the papers published in the last three decades. In fact, 60% of them were published in the last decade [6,7,8,9,10,11,12,13,14,15,16,17,18,19,20,21,22,23,24,25,26,27,28,29,30,31,32,33] and 30% were published in the years 2001–2010 [34,35,36,37,38,39,40,41,42,43,44,45,46,47,48,49,50,51,52,53,54,55,56]. The studies on operational speciation of PTEs dealt primarily with air quality in Europe (40%) [6,10,11,14,19,20,28] and Asia (40%) [7,8,9,12,13,15,16,17,21,22,23,29,30,31,32,33]. The other studies were conducted in the Americas [27,35,43,48,52,56,57] and Africa [18,24,25]. Thus, this review concerns urban areas of different population sizes with low or high industrial activity, with various agricultural influences and with dry and wet climates. In total, 112 separate laboratory data sets published in 70 articles during the last thirty years were compiled and analyzed in this study. The data reflect quite well the current state of affairs regarding the operational speciation of particulate-bound elements determined by sequential extraction methods. The focus was on chemical fractionation results, while the results of the speciation analysis, including valence speciation and physical speciation, were set aside to be discussed separately.

Chemical fractionation of PTEs in atmospheric particulate matter has attracted less interest than chemical fractionation of metals in soils or sediments because of the very real difficulties in measuring low concentrations in low-mass samples. For comparison, only in the last decade the Scopus database registered over 750 articles with the terms “chemical fractionation” AND “metal” AND “soil” in titles, abstracts or keywords, 500 articles with “chemical fractionation” AND “metal” AND “sediment” but only 42 articles with “chemical fractionation” AND “metal” AND “PM”.

The most frequent subject of these studies was a group of toxic metals: As, Cd, Cr, Cu, Fe, Mn, Ni, Pb and Zn (50–90% of the papers) [9,12,13,16,21,27,30,33,34,35]. The elements such as Al [15,18,20,26,32,33], Co [9,12,14,17,21,23,26,27,28,29,30], V [23,26,27,33,57,58,59,60], Ti [10,19,20,23,26,27,32,33], Sb [10,11,14,20,27,28,32], Mo [20,32,33,59,60,61], Se [11,14,28,47,55,56,62,63] and Sn [11,14,28,36,47,56,62] were studied less frequently (10–40%). The study of operational speciation of Ag [18,61], Hg [11,14], Pt [59,60,61], Rh [60], Sc [60], Tl [37,46], Te [61,62] and Bi [59,61] was performed sporadically. The scope of the research presented in a single publication ranged from a few to roughly a dozen elements, with an average of nine covered per paper. Only a few publications were focused on a single element: As [6,7,8] and Pb [64].

Among all PTEs, Cd, Cr, Cu, Fe, Mn, Ni, Pb, V and Zn are the metallic elements of the most immediate concern because of their detection frequency. Exposure to these metals has been associated with cardiovascular diseases, cancer and many other adverse health effects, and their specific effects within different age groups vary widely [3,9,65,66].

Taking the above into account, the particular aims of this work were formulated as follows: to develop average distribution patterns of chemical forms for ten PTEs (As, Cd, Co, Cr, Cu, Fe, Mn, Ni, Pb and Zn) occurring in airborne particles worldwide; and to develop inhalation risk predictions for city dwellers breathing air that is moderately or extremely polluted with these elements. The complementary goals were the assessment of the uniformity of the terminology and the laboratory procedures used.

## 2. Terminology

Even a superficial reading of the publications leads to the conclusion that the International Union of Pure and Applied Chemistry (IUPAC) Recommendations from 2000 have not been fully implemented [67]. The terms: “speciation analysis”, to refer to the activity of identifying and measuring species, and “speciation”, meaning the distribution of an element among defined chemical species, are commonly used. However, dissemination of symmetrical definitions of fractionation still remains a rather distant goal. It is worth recalling that IUPAC Recommendations were made to restrict the use of the term fractionation to refer to analytical activities. A scan of 70 peer-reviewed articles on fractionation of PTEs in APMs published over the last 30 years reflects various approaches in this regard. The authors fractionated the elements taking into account both the chemical bond and the particle size. To specify the type of analytical activity, the term “chemical fractionation” was often used for fractionation by chemical bonding via sequential extraction procedures [8,10,11,12,13,14,15,34,35,36,37,38,63,68,69,70]. One may also encounter terms that are equivalent to the term “speciation analysis”, i.e., “fractionation analysis” [11,39] or “chemical fractionation analysis” [37]. The term “fractionation” was also used in the sense of the distribution of forms of an element [16,17,18,19,71]. However, for this purpose, the authors much more often used other terms: “speciation” or “chemical speciation” [6,10,18,20,21,22,34,39,40,58,69,71,72,73,74], “solid-state speciation” [41,75,76] and “operational speciation” or “operationally defined speciation” [19,42,77]. In this review paper the term “chemical fractionation” is used exclusively for analytical activities, whereas the distribution of chemical forms of a certain element is defined in this paper as its “operational speciation” or the “distribution pattern of its chemical fractions” (also geochemical forms and operationally defined fractions). The lack of a uniform terminology also relates to the research subject. In the set of publications reviewed, the research subject was defined as “heavy metal(s)” [13,16,17,23,43,71], “trace metal(s)” [24,25,34,41,44,75] and “trace element(s)” [10,11,14,26,27,45]. All three terms appeared in similar numbers. In the titles of those articles, the term “heavy metals” occurred more often than “trace metals” or “trace elements”, whilst in keywords it occurred twice as frequently as these other terms. The terms which were proposed to replace the criticized but commonly used term “heavy metals” [78,79] were rarely used: “potentially toxic metals” [13,17,19,20,68,69,80], “potentially toxic elements” [19,46,69], “potentially toxic trace elements” [11,63] and “potential hazardous elements” [70]. In our work, we wanted to respond positively to these terminological proposals and therefore all particulate-bound elements for which chemical fractionation data have been published are described as “potentially toxic elements” (PTEs).

## 3. Chemical Fractionation Procedures

In the reviewed articles, chemical fractionation of PTEs was performed on samples of different grain fractions of atmospheric aerosols: total suspended particulate fraction (TSP)—28%, PM10—26% and PM2.5—37% of the total number of studies. Occasionally, studies were also carried out on PM_7_ [43], PM_1_ [6,11,14,42] and PM_0.6_ [42] samples. The airborne particulate matter was collected on quartz fibre filter membranes (38%), PTFE filter membranes (29%), fibreglass filters (18%) and others (e.g., cotton paper filters, cellulose ester filters, polycarbonate membrane filters). Before sampling, the membrane filters were conditioned in a desiccator. Quartz filters were sometimes additionally pre-heated at 450–800 °C to eliminate organic contamination [13,22,23,34,50,61,69,74], while cellulose filters were occasionally soaked in ultra-pure HNO_3_ or HCl to reduce blank concentrations of metallic elements [32,72]. Field blank filters were collected to reduce gravimetric bias due to filter handling during and after sampling [30,80]. APM samples were collected at the height of 1–6 m [14,21,25,28,43,45,61,64,69,77], 12–20 m (roofs of buildings) [9,16,23,34,44,47,64,80] and in a few cases even at 30–35 m [76,81]. Low-volume and high-volume samplers were used exclusively, which, for the most frequently collected 24 h aerosol samples, was associated with the flow of several dozen or several thousand cubic meters of air. It is worth pointing out that some of the more general descriptions of sampling did not contain such detailed information.

**Figure 1:**
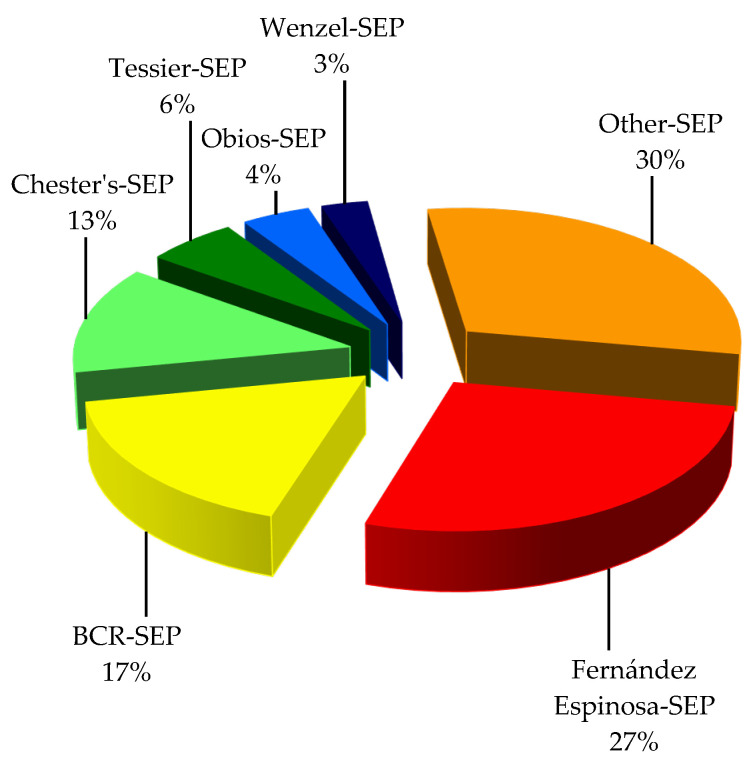
Sequential extraction procedures used for chemical fractionation of potentially toxic elements in airborne particles.

Many sequential extraction methods have been reported, but the procedure developed by Fernández Espinosa [40] turned out to be the most widely used to evaluate the possible chemical associations of PTEs with different geochemical fractions of APM—27% of the surveyed papers (Figure 1).

The procedures that were used less often are the BCR procedure [82,83]—17% of the papers—and Chester’s procedure [75]—used in 13% of the papers. The remaining sequential extraction procedures were employed occasionally: the Tessier SEP [84]—6%, the Obios SEP [85]—4% and the Wenzel SEP [86]—3%.

Alternative sequential extraction procedures comprising two, three or four stages were proposed by authors to accomplish a specific research aim—30% of the papers in total. The above statistics do not mean that the SEPs that were used followed the precise methodologies outlined in the resource articles (Table 1).

The Fernández Espinosa procedure in its original form was used only in 40% of the papers. In the remaining studies this procedure included a number of changes with a potentially different impact on chemical fractionation results. Ultrasound-assisted extraction at both RT [17] and at 70 °C [15] was used to reduce the time of extraction of the soluble and exchangeable fraction from 3 h to 30 min. It is worth mentioning the results of the studies carried out by Desboeufs et al. (2005), who demonstrated that 90% of the total dissolved content of individual metals is always released in the first 20 min of dissolution [87]. Modifications of the release of the carbonate and oxide fraction mainly consisted in reducing the extraction time from 5 h to 4 h [48], to 2 h [17,18,24] and to 20 min, but under ultrasound-assisted conditions [15]. In the same way Wu et al. (2021) accelerated the third step, from 90 min shaking at RT to 20 min shaking in an ultrasonic water bath also at RT [15]. The most serious modifications concerned the extraction of the residual fraction. The introduction of HF into the acid mixture, variously HNO_3_, HCl, HF [15,17,18,24], HNO_3_, HF, H_2_O_2_ [20] and HNO_3_, HF, HClO_4_ [48], resulted in an increase in the range of chemical fractionation from the pseudo-total content of PTEs to their total content.

By using microwave-assisted or ultrasonic assisted extraction, it was possible to accelerate digestion from 5 h to 20 min [15,18,24,48]. Microwave-assisted extraction was also used to digest APM samples in a typical mixture of acids (HNO_3_, HCl, HClO_4_) [29,30]. Conventional acid digestion of the residual fraction was carried out also under milder conditions e.g., at RT [10,11,28] and under more extreme conditions e.g., at 180 °C [34] than those proposed in the source procedure (95 °C). Chemical fractionation of atmospheric particle-bound metallic elements was carried out using two BCR procedures, according to the original BCR sequential extraction protocol [82] and to the modified BCR protocol [83]. Only in a few studies did the fractionation procedure accurately adhere to the developed protocols [71,73]. A reduction in the fractionation time was achieved using microwave-assisted extraction [58,63,74] or ultrasound-assisted extraction [39]. The largest range of modifications concerned the digestion of the residual fraction. Instead of the recommended *aqua regia*, other mixtures of concentrated acids were used, comprising HNO_3_, HClO_4_, HF [19], HNO_3_, HClO_4_, HCl [21], HNO_3_, H_2_O_2_ and HF [63] or only concentrated HNO_3_ [74]. The digestion process was carried out both under milder and under more drastic conditions than the recommended ones: e.g., RT for 30 min [31] or 90–160 °C for 16 h [19] or 190 °C for 24 h [72].

The described modifications of Chester’s procedure involved the addition of an extra stage (water extraction) to the three-stage SEP [12,35,49] and a change in the digestion mixture composition [12,35,49]. The release of the water-soluble fraction was carried out during a short (15 min) shaking at RT [35,49] or a longer extraction (60 min) assisted by ultrasound [12]. In order to ensure the complete dissolution of residual particles, concentrated HCl [49] or 30% H_2_O_2_ [12,35] were added to the mixture of HNO_3_ and HF acids. Acid digestion was performed conventionally as in the original procedure [41,50,51,75,76,81] or by using microwave-assisted extraction [12,35].

The Tessier procedure was employed relatively infrequently [9,25,45,52]. The introduced modifications consisted in determination of the water-soluble fraction of PTEs prior to the conventional procedure [45,52] and in the combined release of the carbonates fraction and the Fe/Mn oxides fraction by leaching with NH_2_OH∙HCl/25% AcOH solution at 96 °C for 6 h [25]. The Obios procedure was used only according to the original version [53,77,85]. In the studies on fraction distribution of arsenic in APM samples [7,8], the chemical fractionation was carried out according to the Wenzel procedure [86].

It is worth emphasizing that although the modifications of the original SEPs undoubtedly have some justification, in practice, each change to an operationally defined procedure makes it still more difficult to compare the results obtained in different laboratories for samples originating from distinct geographical areas or even from similar urban centers. 

**Figure 2:**
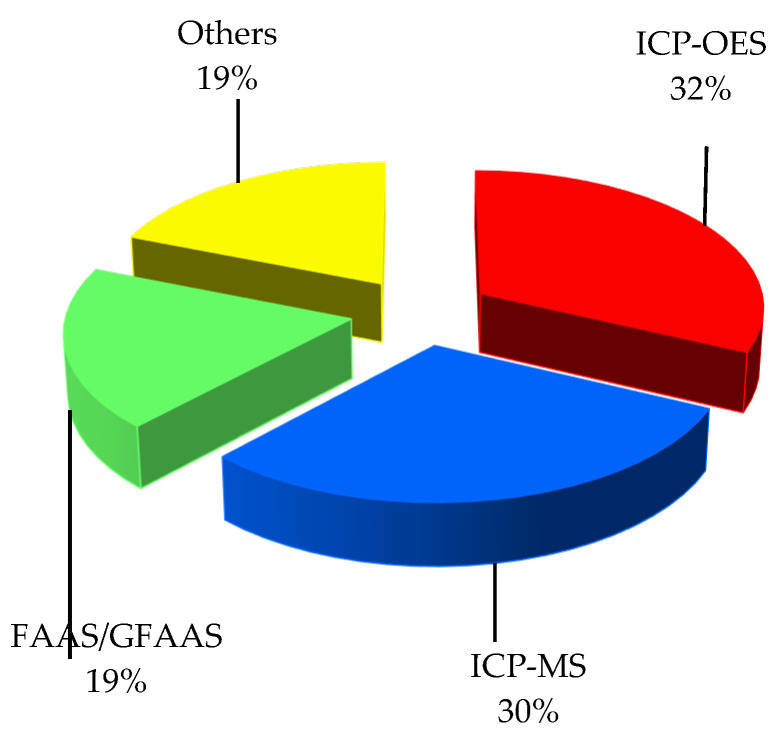
Analytical techniques used for the determination of metallic and metalloid elements in digests and extract solutions of APM samples.

Metal and metalloid elements in sequential extracts and digests were determined using the following instrumental techniques: inductively coupled plasma optical emission spectrometry (ICP-OES or ICP-AES); inductively coupled plasma mass spectrometry (ICP-MS); atomic absorption spectrometry using either a flame or a flameless mode depending on concentration level (FAAS and GFAAS); energy dispersive X-ray fluorescence spectrometry (EDXRF); atomic fluorescence spectrometry (AFS); capillary electrophoresis (CE); or differential pulse anodic stripping voltammetry (DPASV). The three most commonly applied techniques were ICP-OES—32%, ICP-MS—30% and FAAS/GFAAS—19% of the papers (Figure 2).

The other techniques were used in the quantitative elemental analysis by individual research teams, as follows: EDXRF [62], DPASV [45] and CE [39], with AFS used only for the determination of arsenic [7,8]. Since ICP-OES and ICP-MS do not yield the same reliable results for all investigated elements, multi-element analysis was performed in many speciation studies using both these techniques [13,16,19,20,31,37,71] or ICP-OES and GFAAS [41].

It is worth noting that the determined PTE concentrations were always corrected using their average blank concentrations (blank filter, reagents and vessels). Since standard reference materials regarding the chemical fraction dependent composition are not available, validations of chemical fractionation procedures were carried out using various other reference materials, as follows: VKI (QC loam soil A); PACS-2 (marine sediment) [49]; ERM-CZ120 (fine dust) and SRM 1648a (urban particulate matter) [11,14,26]; SRM 1648 (urban particulate matter) [47,48,55,56]; CRM BCR-701 (lake sediment) [19,25,26,48]; and SRM 1633b (coal fly ash) [27,56]. The metallic elements recovery of sequential extraction procedure was enhanced by comparing the sum of all the chemical fraction concentrations with their certified total concentrations. In turn, an internal check of the recovery of metallic elements by the SEP was performed by comparing the sum of all the chemical fractions with the total contents of these elements in examined samples [13,16,17,21,22,29,33,70,71]. It was considered that the SEP was reliable and repeatable if the recovery range was 85–115% for studied elements [13,16,17,22,29,33,70].

## 4. Operational Speciation of As, Cd, Co, Cr, Cu, Fe, Mn, Ni, Pb and Zn

The distribution patterns of the 10 elements in airborne particles were developed for the three most frequently used procedures: the Fernández Espinosa SEP, the BCR SEPs and Chester’s SEP. All literature data regarding operational speciation used in this review are shown in Figures S1–S10. Individual data sets for a specific element and a given SEP were obtained in various laboratories and for various APM samples. Mean concentrations of most of these elements in the air were of the same order of magnitude in the groups defined by a given procedure of chemical fractionation. Statistical analysis of these literature data sets is shown in Table 2, Table 3 and Table 4. In general, the results (percentage of a chemical fraction) show high dispersion, since two thirds of the variation coefficient values are above 50%. It can be observed that 75% of the skewness coefficients are positive, and therefore the percentage fraction values are grouped around the values below the mean percentage (positively skewed distribution). The near zero skewness coefficient (0.0 ± 0.3) applies only to a small amount of data, and most often symmetry describes the percentage of the F(1) fraction, e.g., Cd, Co, Cu and Zn. The kurtosis does not show dominant tendencies; for each of the three data sets, the number of negative values is equivalent to the number of positive values with few but significantly high values of the kurtosis being positive. It is worth emphasizing that the kurtosis values for more than half of these data are in the range of ±1, which indicates a normal distribution of the data.

The most commonly used Fernández Espinosa scheme enables the division of the mobilizable content of potentially toxic elements into four fractions: F(1)—water-soluble and exchangeable; F(2)—carbonate, oxides and reducible; F(3)—bound to organic matter, oxidizable and sulfidic; and F(4)—residual fraction. Chemical distribution patterns of the 10 elements in airborne particles were compiled from 11–23 (av. 18) sets of data obtained from various urban areas from around the world (Table 2).

A distinctive feature of the occurrence of PTEs in APM was the significant content of the water-soluble and exchangeable fraction. The highest percentage is achieved by Cd (42.5%), whereas Co (35.7%), Zn (32.3%), Mn (30.8%) and As (29.2%) are characterized by a slightly lower level of the mobile fraction. The percentage of the water-soluble fraction does not drop below 20% for Cu, Ni and Pb. Only in the case of iron and chromium, the water-soluble fraction binds a small percentage of these metallic elements: 6.5% and 14.6%, respectively. Low mobility of these elements is also confirmed by a high proportion of the residual fraction: 51.0% and 46.0%, respectively.

The compilation of chemical distribution patterns of heavy metals according to the BCR schemes (original and optimized) was developed on the basis of 8–20 (av. 15) data sets from chemical fractionation of particulate matter samples (Table 3). Using the BCR SEP, it is possible to divide the content of metallic elements into four operationally defined fractions: acid-soluble fraction, reducible fraction, oxidizable fraction and residual fraction. In the case of five elements (As, Cd, Cu, Pb and Zn), the dominant fraction was the most mobile fraction—the acid-soluble fraction. As 0.11 M AcOH is a more “aggressive” reagent than water, it was expected that the mean percentage of the acid-soluble fraction should not be lower than the mean percentage of the water-soluble fraction. In fact, only in the case of Co and Ni did the percentage of the acid-soluble fraction (BCR SEP) prove to be lower than the percentage of the water-soluble and exchangeable fraction (F-E SEP).

Operational speciation of the selected PTEs according to Chester’s scheme was developed on the basis of the published 7–15 (av. 12) data sets from chemical fractionation of urban-APM samples (Table 4). This chemical fractionation procedure enables the division of the content of PTEs into three fractions: loosely held fraction; carbonate and oxide fraction; and refractory and organic fraction. Although formally the fraction F(3) in this scheme can be treated as a sum of two fractions, F(3) and F(4), in the Fernández Espinosa and the BCR schemes, it should not be forgotten that the differences in the procedures of their release are significant (Table 1).

In spite of the fact that the results of the sequential extraction of environmental solid samples depend on the scheme used for their determination, an attempt has been made to compare the results of the chemical fractionation of PTEs in APM by using the Fernández Espinosa, BCR and Chester procedures. The evaluation focused on the percentages of individual chemical fractions. The similarity level turned out to be small. Irrespective of the procedure used, only Cd shows a high percentage of the F(1) fraction. A moderate proportion of this fraction (22.6–37.8%) can be found in the case of As, Cu and Mn, while the percentage of F(1)-Fe is definitely low: 6.5% (F-E SEP), 9.8% (BCR SEPs) and 6.1% (Chester’s SEP). The percentage values of F(2) make it impossible to conduct an analogous evaluation, whereas a similar proportion of the residual fraction concerns only Fe: 51.0% (F-E SEP), 47.9% (BCR SEPs) and 65.7% (Chester’s SEP).

**Figure 3:**
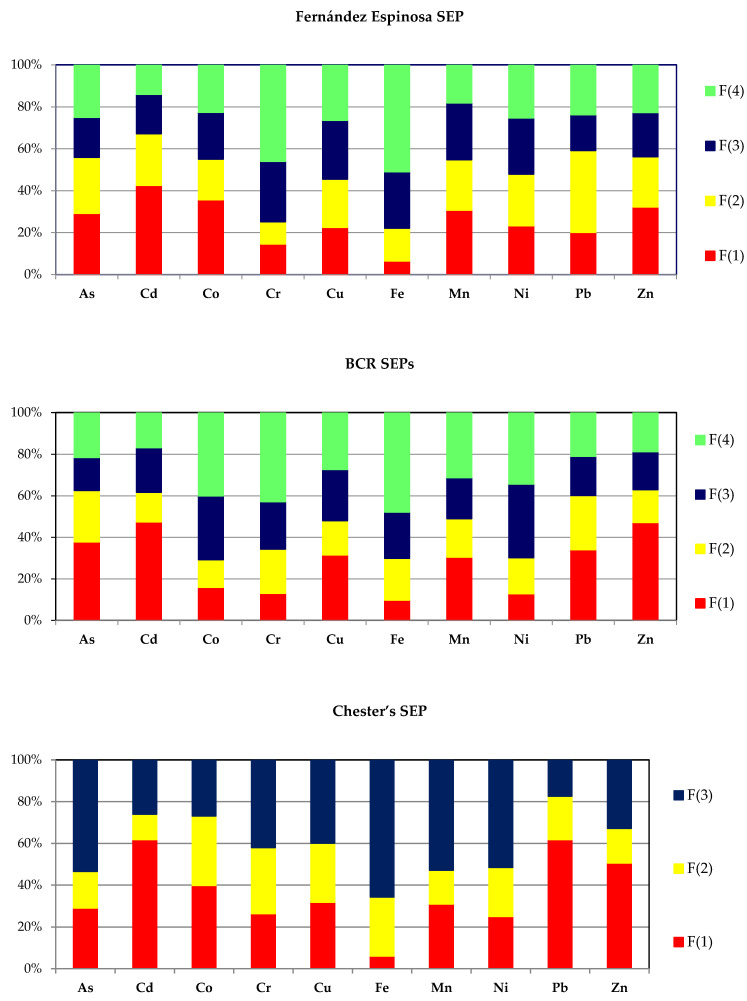
Average operational speciation of selected PTEs in the urban air worldwide.

The mean operational speciation of selected PTEs in the urban air worldwide is given in Figure 3.

Operational speciation of individual elements is as follows:

Arsenic

The chemical distribution of As performed by F-E SEP is relatively balanced (Table 2). The percentages of fractions F(1)-As, F(2)-As and F(4)-As are quite similar (29.2%, 26.7% and 25.0%, respectively). Only the percentage of the organic, sulfidic and oxidizable fraction is lower—19.2%. The patterns of chemical distribution of As by the BCR SEPs (Table 3) and Chester’s SEP (Table 4) are different. The speciation studies using the BCR method have shown that As is present mainly in the acid-soluble fraction (37.8%), while the results obtained by means of Chester’s procedure show that As is mainly bound to the refractory and organic fractions (53.5%). The view that the important chemical species of As in urban APM are As_2_O_3_, As_2_S_3_, As_2_O_5_, As_2_S_5_ and organoarsenic compounds [48,51] is not divergent from these results.

Cadmium

Cd is one of the metallic elements for which operational speciation is almost independent of the applied fractionation procedure (F-E SEP, BCR SEPs or Chester’s SEP). Cd is mostly accumulated in the most mobile fraction F(1): 42.5%, 47.3% and 61.8%, respectively. The residual fraction bound only 14.0–16.9% of Cd (F-E SEP and BCR SEPs). In Chester’s scheme, an analogous fraction is not determined.

From among the cadmium species detected in aerosols (Cd, CdCl_2_, CdO, Cd(OH)_2_, CdS and mixed oxides with Cu and Zn) [16,51], the F(1)-Cd fraction should be linked primarily with the presence of CdCl_2_, although CdO and Cd(OH)_2_ may also be present.

Cobalt

Distribution of chemical forms of cobalt shows a high share of the water-soluble fraction (35.7%) and an even partition between the other three fractions (F-E SEP). The distribution pattern developed on the basis of the results of the chemical fractionation by Chester’s procedure is similar, while the operational speciation of Co based on the results of the chemical fractionation according to the BCR scheme is different in character. The residual fraction is dominant (40.0%), whereas mobile fractions F(1)-Co and F(2)-Co bind only 15.9% and 13.3% of cobalt, respectively.

Chromium

Chromium shows low mobility and is characterized by a large proportion in the residual fraction: 46.0% (F-E scheme), 42.8% (BCR-scheme) and 42.1% (Chester’s scheme), and a relatively low proportion in the F(1)-Cr fraction: 14.6% (F-E scheme), 13.0% (BCR scheme) and 26.4% (Chester’s scheme). The relatively high percentage of the F(1)-Cr fraction in the data set of Chester’s scheme can be easily explained by the use of an extractant with strong complexing properties of Cr(III)—1 M AcONH_4_.

Copper

Copper occurring in urban APM is characterized by an exceptionally uniform distribution among four chemical fractions regardless of the chemical fractionation procedure used: F-E SEP or BCR SEP. The even partition among three chemical fractions also refers to operational speciation by Chester’s SEP. Due to considerable interest in copper speciation in APM, there is relatively extensive literature on the identification of Cu species present in individual fractions. The water-soluble and exchangeable fraction is explained by the presence of CuCl_2_, CuSO_4_ and Cu(NO_3_)_2_ [16,22,27,45,72]. The organic, sulfidic and oxidizable fraction comprises sulfide species such as Cu_2_S, CuS, CuFeS_2_ and Cu_5_FeS_4_ [16,47,48]. The immobile residual fraction is formed by Cu bound to silicates [27,47] and magnetite with a significant Cu content emitted both by steel metallurgical industry and by vehicle traffic [19].

Iron

Iron is an element with the least mobility and its operational speciation is characterized by a significant diversity of distribution. The percentage of iron bound to individual chemical fractions increases, regardless of the chemical fractionation procedure used, in the following order: F(1) = 6.5% < F(2) = 15.7% < F(3) = 26.9% < F(4) = 51.0% (F-E SEP), F(1) = 9.8% < F(2) = 20.1% < F(3) = 22.3% < F(4) = 47.9% (BCR SEPs) and F(1) = 6.1% < F(2) = 28.2% < F(3) = 65.7% (Chester’s SEP). The water-soluble fraction of iron is linked with the presence of iron sulfate, nitrate and/or oxalate [16,48]. Possible compounds contained in the F(2)-Fe fraction are Fe_2_O_3_, FeO(OH) and FeCO_3_ [16,48], while magnetite, hematite and iron species in clay mineral can be present in the residual fraction [20,39,47,48].

Manganese

Chemical distribution patterns of Mn developed for three different data sets (F-E SEP, BCR SEPs and Chester’s SEP) are very similar. The percentage of F(1)-Mn is the same in each of the distribution patterns: 30.8%, 30.5% and 31.0% (Table 2, Table 3 and Table 4). The overall proportions of the refractory and oxidizable fractions are also similar: 45.2%, 51.0% and 52.9%. Mn may occur as MnCl_2_ and MnSO_4_ in the soluble and exchangeable fraction [16]. MnO_2_, MnCO_3_ [16] and MnFe_2_O_4_ [48] can be components of the reducible fraction. MnS is assigned to the organic matter and sulfide fraction whereas Mn present in silicates and aluminosilicates is included in the residual fraction [48].

Nickel

Ni, like Cu, is very evenly divided among the four fractions of the F-E scheme. The distribution pattern of Ni derived from a data set for the BCR scheme is different. Of the total Ni, 35.5% and 34.3% is accumulated in the oxidizable fraction and the residual fraction, respectively. Operational speciation of Ni developed on the basis of Chester’s scheme is very similar to the speciation of Ni by the Fernández Espinosa scheme. There is a view that the source of the water-soluble and exchangeable fraction is mainly NiSO_4_, which is derived from coal combustion, while the carbonate and reducible fraction of Ni is formed by NiO and/or more complex oxides [16,29,43].

Lead

Pb(II), similar to Cr(III), very readily forms acetate complexes under the extraction conditions of stage one of Chester’s scheme. Extraction with 0.11 M AcOH solution or only with water is considerably less efficient: 34.0% (BCR SEPs) and 20.1% (F-E SEP) compared with 61.8% (Chester’s SEP), meaning the lower the complexing power, the higher the percentage of Pb determined as the carbonate and oxide fraction: 39.0% (F-E SEP), 26.1% (BCR SEPs) and 20.8% (Chester’s SEP).

The exchangeable and water-soluble fraction may contain soluble lead halides (PbCl_2_, PbClBr, PbBr_2_ and PbBrCl·2NH_4_Cl) [16]. Less soluble Pb species, such as PbS, PbO, PbSO_4_, PbCrO_4_ and PbCO_3_, are considered to be components of the carbonate fraction or reducible fraction [16,20]. However, on the other hand, Funasaka et al. (2013) report that Pb^0^, PbSO_4_, PbSiO_3_, PbS and PbO_2_ are mainly extracted with the residual fraction [64].

Zinc

The chemical distribution pattern of Zn reflects the mobile nature of this element. Most Zn is found in the F(1)-Zn fraction, regardless of the fractionation procedure used to study its chemical distribution: 32.3% (F-E SEP), 47.1% (BCR SEPs) and 50.6% (Chester’s SEP). A considerably lower proportion of Zn is in the residual fractions (F-E SEP and BCR SEPs) or the refractory and organic fraction (Chester’s SEP): 22.8%, 18.7% and 32.8%, respectively.

The water-soluble fraction of Zn is mainly formed by ZnSO_4_ and ZnCl_2_ [16,19,27,47], whereas the carbonate fraction or the reducible fraction mostly comprises ZnO and carbonates e.g., CaCO_3_·ZnCO_3_ [16]. In the residual fraction, Zn may be bound with Fe and Mn oxides, aluminosilicates and silicates [20,27,47].

**Figure 4:**
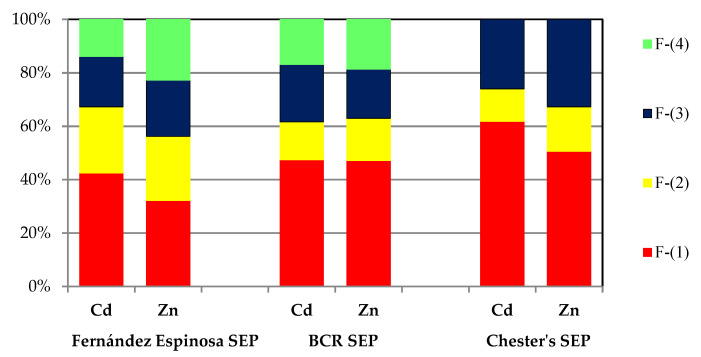
Comparison of the average operational speciation of cadmium and zinc in atmospheric particulate matter.

A detailed analysis of the reported results of the chemical fractionation of Zn and Cd leads to the conclusion that each of the three procedures (Fernández Espinosa SEP, BCR SEP and Chester’s SEP) offers equivalent possibilities for studying operational speciation. Comparable chemical distribution patterns were obtained for elements with similar chemical properties (the same subgroup of the periodic table—the zinc family) (Figure 4).

## 5. Water-Soluble Fraction of Potentially Toxic Elements

It is assumed that the water-soluble fraction of PTEs in APM contains free metal ions and metal ions complexed with soluble organic substances [4]. An increase in ionic strength caused by the most rapidly dissolved metal species (chlorides, sulfates, nitrates, acetates, etc.) can also contribute to a release of exchangeable metal species [40]. Soluble PTEs are generated mainly from industrial sources (50–70%) [27]. The metals associated with the water-leachable fraction are considered mobile in the environment and bioaccessible to organisms, and therefore represent a potentially harmful component of atmospheric particles. The release of metal elements from atmospheric PM has an important impact also on aqueous atmospheric chemistry and the toxicity of wet deposition, especially in surface waters, because dissolved fractions of metal elements are directly accessible for the phytoplankton [88]. Water extraction is considered the simplest and most universal extraction procedure in toxicity testing by many authors [44]. Since the total or pseudo-total content of PTEs is usually also determined, the whole of the analytical process, regardless of whether the extractions were carried out sequentially or in parallel, allows for the establishment of the distribution of PTEs between the water-soluble fraction and the much less environmentally mobile fraction [47]. This approach to chemical fractionation is considered a good compromise between costs, analytical times and achievable information [54].

In this work, the water solubility data set of ten metallic and metalloid elements included the results of the water extraction of APM samples that were obtained in a two-step extraction procedure [6,27,28,32,44,46,47,59,60,61,64,80] or as the first step in recognized sequential extraction procedures, e.g., Fernández Espinosa SEP [40], modified Tessier SEP [52] and in other SEPs [45,49,55,62]. This data set also includes the results of extraction with 1 M MgCl_2_ and 1% NaCl solutions obtained by using the Tessier SEP and the Obios SEP [9,25,53,77,85]. The water-soluble proportion is expressed as the percentage of the total or pseudo-total concentration of those ten elements. The literature set of ordered pairs (concentration in air, percentage of the water-soluble fraction) has been split into two groups: “Commonly encountered pollution level” (CEPL) and “High pollution level” (HPL). The division was performed fairly arbitrarily, taking into consideration the values of PTE concentrations, air quality standards set by WHO and EC and an intention to build up a CEPL data set as large as possible, but without significant outliers. As a result, the CEPL set for individual elements has from 18 to 60 literature datapoints (average subset size of an element—34 values) while the HPL set has from four to 24 literature datapoints (average subset size of an element—11 values). The data set of CEPL is considered by us to be characteristic of an urban environment and thus the basic data set.

The statistical properties of this data set are provided in Table 5.

Generally, low skewness values justify the use of an arithmetic mean as a measure of central tendency (only two skewness values are clearly greater than 1). The mean percentages of water-soluble fractions are in most cases close to their corresponding median values, while the mean values of concentrations of most PTEs are slightly higher than the corresponding median values (typical of a positively skewed normal distribution, which is characteristic of naturally occurring phenomena). The negative kurtosis values (platykurtic distribution) indicate the absence of outliers and a greater clustering of results around the mean. The exceptions are the large kurtosis coefficients for the concentrations of Co and Ni.

Even a cursory assessment of the collected literature data reveals considerable differences among concentrations of individual PTEs in the urban air. For metal concentrations classified as CEPL, Co and Cd have the lowest mean concentrations—1.2 ± 0.5 ng/m^3^ and 3.3 ± 0.5 ng/m^3^, respectively (Table 5). The mean concentration of Cd is below the EU target value (5 ng/m^3^) [89]. Slightly higher levels can be found for the concentrations of Cr (13 ± 2 ng/m^3^), Ni (16 ± 4 ng/m^3^) and As (19 ± 3 ng/m^3^) but the concentration of Ni is below the EU target value of 20 ng/m^3^, whereas the concentration of As exceeds the EU target value (6 ng/m^3^) threefold [89]. The mean concentration of Mn (38 ± 6 ng/m^3^) is considerably lower than the WHO guideline value of 150 ng/m^3^ [91]. The highest values are exhibited by the mean concentrations of Fe (4.6 × 10^2^ ± 0.7 × 10^2^ ng/m^3^), Pb (1.3 × 10^2^ ± 0.2 × 10^2^ ng/m^3^) and Zn (2.6 × 10^2^ ± 0.4 × 10^2^ ng/m^3^). It is worth pointing out that the mean concentration of Pb is well below the EU limit value (500 ng/m^3^) [90]. In urban areas classified by us as extremely polluted, mean concentrations of TMEs are one to two orders of magnitude higher. Against this background, air pollution with toxic trace metals in the EU area looks more favourable. In 2018, from among 700 monitoring stations located in 28 European countries, only a few reported concentrations of As, Cd, Pb and Ni above the target values, and 95–98% of the stations reported As, Cd, Pb and Ni concentrations below or equal to the lower assessment threshold (LAT): 2.4 ng/m^3^, 2 ng/m^3^, 250 ng/m^3^ and 10 ng/m^3^, respectively [91].

**Figure 5:**
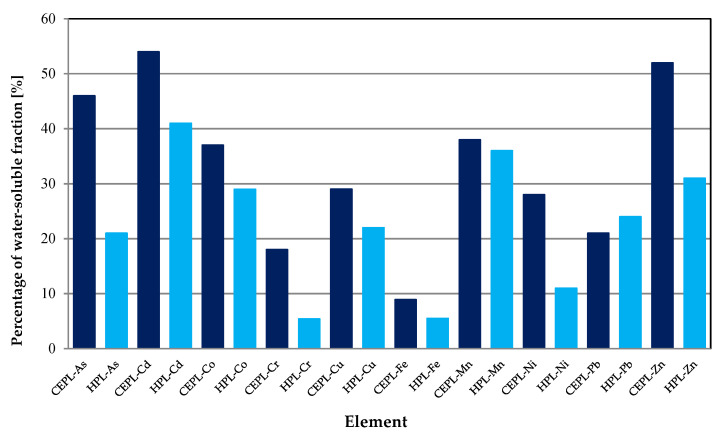
Mean proportion of water-soluble fractions of PTEs in the urban air classified as follows: CEPL—commonly encountered pollution level; HPL—high pollution level.

Despite such significant differences in concentrations of PTEs in the urban air, it was discovered that the proportion of the water-soluble fraction of these elements was not subject to large fluctuations (Figure 5).

Generally, PTEs reaching extremely high concentrations in the urban air are characterized by lower proportions in the most mobile fraction. Only the percentage of the water-soluble fraction of Mn and Pb remains at a stable level, irrespective of its concentration in air. The greatest reduction in water solubility was observed for Cr, for the CEPL—18% and for the HPL—5.5%. In the moderately polluted urban air, the order of water leachability of the PTEs was as follows: Cd ≈ Zn > As > Co ≈ Mn > Cu ≈ Ni > Pb > Cr > Fe. This was different from the order of the water leachabilities of these elements determined for highly polluted urban areas: Cd > Mn > Zn > Co > Pb > Cu ≈ As > Ni > Fe ≈ Cr. However, these differences are not large. For the data set belonging to the CEPL, Cd, Zn and As have the highest proportion in the water-soluble fraction: 54%, 52% and 46%, respectively. The percentage of the water-soluble fraction of Mn, Co, Cu and Ni is lower, but still relatively high (from 28% to 38%). Only Fe is characterized by a low proportion in the water-soluble fraction (8.9%). Water extractions are very often used to assess the bioaccessibility of PTEs. Thus, according to the classification proposed by Mbengue et al. (2015) [20], the analyzed PTEs can be divided into three groups depending on their potential bioaccessibility for CEPL:

(1) not very bioaccessible (<20%): Fe and Cr;
(2) moderately bioaccessible (20–40%): Pb, Ni, Cu, Co and Mn;
(3) highly bioaccessible (40–60%): As, Zn and Cd.

For HPL, on the other hand, the PTEs can be divided as follows:

(1) not very bioaccessible: Cr, Fe, and Ni;
(2) moderately bioaccessible: As, Cu, Pb, Co, Zn, and Mn;
(3) highly bioaccessible: Cd.

Due to dissimilar water solubilities, the water-soluble concentrations of elements may differ from the sequence of their concentrations in APM [32]. Hence, taking into account the concentration of the water-soluble fraction of these elements in the air, it is Zn, Pb, Mn and Cu that pose the highest potential risk of getting into human bodily fluids and then into the bloodstream. Notable aspects of the research on atmospheric operational speciation also include the effects of seasonal changes and size distribution of PM on PTE water-solubility. On the whole, a proportion of the water-soluble fraction of PTEs was higher in a fine fraction than in a coarse fraction of atmospheric particles [12,32,44,47]. The differences are not significant, although a few exceptions have been found, e.g., Ni (4×, Edinburgh, UK) [44] and Cu (10×, Pune, India) [12]. The study of seasonal variations in the water-soluble proportion usually includes four seasons; however, due to emissions from fossil fuel combustion, winter was often the season with the highest percentage of metals in the water-soluble fraction [6,15,32,33,48,61]. Due to a relatively small number of reports, the latter observations do not constitute a sufficient basis for broader generalizations.

## 6. Environmental Risk Assessment

In order to assess the risk in an environment polluted by PTEs, it is common to use a number of individual and complex indices, the most important being the following: geoaccumulation index (I_geo_); enrichment factor (EF); bioavailability index (BI); individual and global contamination factor (ICF and GCF); risk assessment code (RAC); pollution load index (PLI); degree of contamination (C_deg_); and potential ecological risk (RI) [12,16,92]. Some indices are defined by the total metal concentration (e.g., I_geo_, EF and PLI) and others by a concentration of a defined fraction of these metals (e.g., BI, RAC and ICF). Wider use of these indices beyond a specific research project necessitates the comparison of a set of indicators determined by various research teams. The QA/QC system commonly implemented in analytical laboratories ensures the collection of reliable data on total concentrations of trace elements in environmental samples. However, establishing the traceability of speciation analysis results using operational chemical fractionation procedures is still a challenge for environmental analysts [93].

As mentioned above, individual SEPs applied to a defined analytical task can yield different results. The differences result from a different approach to the kind and sequence of geochemical phases being dissolved and from different conditions of extraction of even the same geochemical phases. Thus, expecting good compatibility of environmental indices determined on the basis of data from multistage chemical fractionation is rather unrealistic. In the case of the procedures that are most frequently used for fractionation of PTEs in APM, a comparison of the results of the first stage of extraction is ineffective due to the different ranges of these extractions: the soluble and exchangeable fraction (Fernández Espinosa SEP), the exchangeable, water- and acid-soluble fraction (BCR SEP) and the loosely held fraction (Chester’s SEP) (Table 1). A similar remark applies to the second stage of these chemical fractionation schemes: the carbonate, oxide and reducible fraction (Fernández Espinosa SEP), the reducible fraction (BCR SEP) and the carbonate and oxide fraction (Chester’s SEP). The common element of these three data sets can be the combined fraction that is the sum of the extraction results of the first and second stages and therefore it can serve as a suitable tool for making comparisons. For a given SEP, the fraction defined in this way includes water-soluble and exchangeable PTEs as well as PTEs bound to carbonates and oxides. Jan et al. (2018) define this combined fraction as a non-resistant fraction of a metallic element that has become available to a human body and/or plants, and the remaining part of this element as its resistant fraction [12]. For elements that are potentially toxic to humans, the percentage of the mobile fractions F(1) and F(2) is of special interest because it determines their bioavailability [68]. The contents of the non-resistant fractions as a percentage of the mean total concentrations of PTEs are shown in Table 6.

All the data concern the results of the chemical fractionation of PTEs in APM samples from the group “Commonly encountered pollution level”. The most similar percentages of the non-resistant fraction were obtained for Mn, Zn, Fe and Cd; the differences between the largest and smallest values were 7.6%, 10.9%, 12.1% and 12.5%, respectively. An individual contamination factor for each of the ten PTEs was determined as a measure of the degree of metal contamination in the environment with respect to a retention time of these elements [94] and the bioavailability index that is used to evaluate the potential mobility and bioavailability of metals [34].

ICF was calculated as the ratio of the content of the non-resistant fraction to the resistant fraction of the metal. PTEs were categorized into four classes of contamination: low contamination (ICF < 1), moderate contamination (1 ≤ ICF < 3), considerable contamination (3 ≤ ICF < 6) and high contamination (ICF ≥ 6) [12]. Metals with a higher ICF have a lower retention time, higher environmental mobility and exert a higher risk to the local environment [16].

On the other hand, BI was calculated as the ratio of the content of the non-resistant fraction to the total content of the metal. BI values were divided into three categories: low bioavailability, when BI < 0.3; medium bioavailability, when 0.3 ≤ BI < 0.5; and high bioavailability, when BI ≥ 0.5 [16].

Regardless of the SEP used, the same class of contamination was determined for four PTEs (Cd, Fe, Ni and Zn), whereas the same bioavailability category was determined only for three PTEs (Cd, Pb and Zn). Generally, the indices calculated based on the results from extraction according to the Fernández Espinosa SEP and the BCR SEP showed better comparability than those from extractions according to the Chester SEP and the Fernández Espinosa SEP, or for the Chester SEP and the BCR SEP. The results of extractions by means of the Fernández Espinosa SEP and the BCR SEPs yielded the same class of contamination in the case of eight elements (As, Cd, Cr, Cu, Fe, Ni, Pb and Zn) out of the ten that were analyzed. The comparability of bioavailability was worse; the same category was determined for only six elements (As, Cd, Cu, Fe, Pb and Zn).

## 7. Health Risk Assessment

Metallic and metalloid elements bound in respirable APM can easily get deep into human lung tissues through breathing. The intake of metals into the human body through inhalation over a long period of time and their accumulation can cause serious health risks [34]. For this reason, many researchers have not only studied the contamination of air with PTEs but have also evaluated their impact on human health (slightly over 30% of the reviewed 70 articles). The human-health risk posed by the PTEs due to inhalation of atmospheric particles was most frequently estimated [10,12,13,14,17,24,33], although health risks from other routes of exposure (ingestion and dermal contact) have also been determined [9,15,18,25,29,31,70]. Since PM2.5 is a stronger risk factor than the coarse part of PM 10 [65], this grain fraction is the most commonly used for characterizing inhalation exposures to particulate matter [8,9,10,13,16,18,30]. The risk calculations less frequently took into account the contents of PTEs in the fractions PM10 [12,80], PM1 [14] or TSP [25,70]. Carcinogenic and non-carcinogenic risks were usually assessed [10,12,13,15,16,17,18,24,31,33,63,71], but there are some publications in which only the carcinogenic risk was calculated [29,34,80].

The human risk assessment was estimated on the basis of the human-health risk model developed by the US Environmental Protection Agency [95,96]. The collection of publications reviewed includes papers that estimated health risks according to the current recommended approach for inhaled contaminants (US EPA 2009, Part F) [8,10,14,16,17,18,33,71], and also according to a previous approach (USEPA 1989; Part A) [9,25,31,34,70,80]. It is worth noting that the earlier approach is still being applied, even in recent publications (2018–2021) [9,12,15,25,31]. This kind of approach is not only a formal matter, because in the case of similar concentrations of toxic elements in the air, risk indicators (Hazard Quotient, *HQ* and Hazard Index, *HI*) determined according to these models differ considerably. Lower indicator values reflecting smaller health hazards are obtained according to the previously recommended risk assessment model [15,16,17,24,25,70].

The health risk assessment was usually conducted only for adults [12,34,58,80] or for adults and children [8,10,16,25,71]. There are also some studies in which the risk was estimated separately for a few groups of children and/or adults. The children were classified into two groups (e.g., I: 1–6 years; II: 6–18 years) [24,29] or three groups: infants (0–1 year), toddlers (0.5–5 years) and children (5–19 years) [17], whereas adults were classified into two groups: males and females [9,17].

The human-health risk posed by particulate toxic elements via inhalation has been estimated on the basis of the following: their total [12,13] or pseudo-total concentration [9,29,30,80]; the concentration of mobile fractions (sum of all fractions except the residual fraction, regardless of the SEP used) [12,29,30]; the concentration of the bioaccessible fraction [10,15,16,17,18,24,33,34]; the concentration of the exchangeble fraction [25]; and the concentration of the water-soluble fraction [29,80].

Among all the chemical fractions of the analyzed elements, the water-soluble fraction is the most readily bioaccessible form [17,46] as it can easily transfer into the dissolved fraction and enter the bloodstream from lung fluids [22,58]. As it is this fraction that has the highest potential to cause adverse health effects [17,63,97], in our work, the carcinogenic and non-carcinogenic risk assessment was performed based on literature data regarding the water-soluble fractions of airborne PTEs. The reference point was the risk calculated for their corresponding total concentration of PTEs. A similar approach to health risk assessment can be found in studies by other authors in which the risk determined for mobile fractions or bioaccessible fractions of PTEs was compared with the risk estimated on the basis of the total concentration of PTEs in the air [12,29,30].

In our work, human-health risk was calculated using a risk assessment model recommended by US EPA (2009, Part F) [95]. The exposure concentration (*EC_i_*) of non-carcinogenic and carcinogenic metals was calculated using Equation (1):

(1) $$EC_{i}=\frac{C_{i}\cdot ET\cdot EF\cdot ED}{AT_{n}}$$

where *C_i_* is the mean concentration of a toxic element in the air [μg m^−3^]; *ET*—exposure time [h day^−1^], *EF*—exposure frequency [days year^−1^], *ED*—exposure duration [years] and *AT_n_* is the averaging time of exposure [h].

A residential scenario could consist of inhalation exposure for up to 24 h per day, up to 350 days per year for 6 to 30 years [95]. In the reviewed publications, the most commonly used parameters for adults were *ED* = 24 years [8,10,13,17,29,33,71] and *ET =* 24 h day^−1^ [8,10,13,16,17,18,29,30,33,71]. Lower values were used far less frequently e.g., *ED* = 20 years [10,30] and *ET* = 8 h day^−1^ [13]. A greater variation relates to the exposure frequency value. *EF* = 350 days per year was most commonly used in risk calculations [8,16,25,30,71], but there are publications in which *EF* has lower values: 250 days per year [10,13] or 180 days per year [17,29,33]. The exposure parameters used in our study are: *ED* = 24 years, *ET* = 24 h day^−1^ and *EF* = 350 days per year (Table 7).

The average time of exposure for carcinogens was calculated as *AT_n_* = 70 years⋅365 days year^−1^⋅24 h day^−1^, while for non-carcinogens this was calculated as *AT_n_* = 24 years⋅365 days year^−1^⋅24 h day^−1^. The water-soluble fraction concentrations and total concentrations of toxic and carcinogenic elements calculated by us as the mean of literature data were used as *C_i_* (Table 5). The data refer mostly to the PM2.5 fraction, e.g., for Pb: PM2.5 (42% of the papers), PM1 (5%), PM10 (25%) and TSP (28%).

The non-carcinogenic health risk for each toxic metal was evaluated by means of hazard quotient (*HQ*) using Equation (2):

(2) $$HQ=\frac{EC_{i}}{RfC_{i}\cdot 1000\;\mu g\;{\mathrm{mg}}^{-1}\;}$$

where *RfC_i_*, is the inhalation reference concentration [mg m^−3^]. The overall potential non-carcinogenic risk posed by more than one toxic element was calculated as hazard index (*HI)* using Equation (3):

(3) $$HI=\overset{n}{\underset{i=1}{\sum }}HQ_{i}$$

Cancer risk (*CR*) was calculated for each carcinogenic element using Equation (4):

(4) $$CR_{i}=EC_{i}\cdot IUR$$

The total cancer risk (*TCR*) posed by more than one carcinogenic element was calculated using Equation (5):

(5) $$TCR=\overset{n}{\underset{i=1}{\sum }}CR_{i}$$

A carcinogenic risk value higher than the upper limit (1 × 10^−4^) suggests that the presence of carcinogens in the air results in a high probability of developing cancer through lifetime exposure, while values below the lower limit (1 × 10^−6^) indicate no significant cancer risk. The acceptable risk range is between 1 × 10^−6^ (1 in 1,000,000) and 1 × 10^−4^ (1 in 10,000) [96]. An *HQ* and/or *HI* below 1 indicates that there is no significant risk of non-carcinogenic effects. If *HQ* and/or *HI* equals or exceeds 1, non-carcinogenic effects might occur.

Non-carcinogenic effects were estimated only for As, Cd, Co, Cr, Ni and Mn, since for the rest of the analyzed elements reference concentrations for inhalation exposure were not evaluated [98]. Carcinogenic effects and *CR* values were calculated only for As, Cd, Co, Cr, Ni and Pb, for which *IUR* values were available.

Risk characterization was conducted both for the residents living in cities with low or moderate PTE pollution (CEPL) and those living in cities with air extremely highly polluted with PTEs (HPL). The estimated potential carcinogenic risks are given in Table 8.

The risks estimated on the basis of the water-soluble fraction of an individual element (As, Cd, Co, Cr, Ni and Pb) for residents of CEPL-cities were within the acceptable risk range (between 1 × 10^−6^ and 1 × 10^−4^) and ranged from 1.1 × 10^−7^ to 1.3 × 10^−5^. The *TCR* = 2.5 × 10^−5^ was also lower than the maximum acceptable limit of 1 × 10^−4^, indicating that the carcinogenic risk was acceptable and amounted to a negligible risk posed by As, Cd, Co, Cr, Ni and Pb. It is worth noting that the *TCR* calculated on the basis of the total concentrations of these elements (8.6 × 10^−5^) was also acceptable (Table 8). For residents of HPL-cities, the inhalation cancer risk based also on water-soluble fractions of individual elements was higher than the maximum acceptable limit only in the case of As (4.3 × 10^−4^) and Co (1.5 × 10^−4^), which may indicate potential carcinogenic risks from these two elements. However, the total cancer risk (*TCR* = 6.8 × 10^−^^4^) exceeded 1 × 10^−4^, suggesting a high probability of developing cancer through lifetime exposure.

The reference value calculated for total concentrations of PTEs was almost six times higher (*TCR* = 4 × 10^−3^). The estimated non-carcinogenic inhalation risks from PTEs are listed in Table 9.

The *HQ* values for Cd, Co, Cr, Ni and Mn for residents of CEPL-cities calculated even on the basis of the total concentration were lower than the safe level (*HQ* = 1) and ranged from 1.2 × 10^−1^ to 7.2 × 10^−1^, indicating no non-carcinogenic risks from each individual element. Only in the case of As was the hazard quotient slightly higher than unity (*HQ* = 1.2). The non-carcinogenic risk estimated on the basis of the concentrations of water-soluble fractions of these metals was, on average, three times lower. The total non-carcinogenic risk was acceptable (*HI* = 1.1) only for calculations based on concentrations of water-soluble fractions.

Much greater non-carcinogenic risks exist for residents of HPL-cities (Table 9). In this case, only Cr (*HQ* = 1.8 × 10^−1^) and Ni (*HQ* = 4.2 × 10^−1^) pose no threat. *HQ* values for As, Cd, Co and Mn exceed the acceptable risk level many times over. *HQ* for arsenic reached as much as 20. In the case of the water-soluble fraction, the non-carcinogenic health risk due to exposure to all analyzed PTEs was very high (*HI* = 34). An even higher *HI* = 1.4 × 10^2^ was obtained when the non-carcinogenic risk was estimated on the basis of the total concentration.

Our calculation results demonstrate convincingly that health risks estimated on the basis of total concentrations of metals may result in an overestimation. A similar view has been presented by the authors of the articles reviewed, who suggest that taking into consideration the bioaccessible form rather than the total content of heavy metals in the air gives a more adequate health risk estimate [17,18,24,33]. It is also worth noting that the toxicological studies have suggested it is the soluble toxic trace element content of APM that is more directly linked to its harmful effects, rather than their total content [100,101].

## 8. Conclusions

- Operational speciation of over 40 potentially toxic particulate-bound elements occurring in urban air has been described in the scientific literature. The most common research subjects were As, Cd, Cr, Cu, Fe, Mn, Ni, Pb and Zn.
- Unification of terminology, the procedure for sampling atmospheric aerosols, sample pretreatment and, above all, a sequential extraction procedure is still a distant goal.
- Most operational speciation studies of potentially toxic elements were carried out using one of these three procedures: Fernández Espinosa SEP > BCR SEPs > Chester’s SEP. However, the declared application of a specific procedure did not always mean that the authors strictly followed its original protocols; both physical and chemical operating parameters were altered.
- The distribution patterns developed for 10 potentially toxic elements in urban air particulate matter for three chemical fractionation schemes should serve as useful benchmarks for future atmospheric speciation studies.
- A distinctive feature of operational speciation of particulate-bound metallic elements is the large proportion of the most mobile fraction. From among 10 elements (As, Cd, Co, Cr, Cu, Fe, Mn, Ni, Pb and Zn) present in urban air, Cd and Zn have the highest proportion of this fraction, whilst As, Co, Cu, Mn, Ni and Pb have a moderate proportion, and only Fe and Cr have a relatively low proportion, of this fraction.
- Ecological risk indices calculated on the basis of the results obtained from chemical fractionation according to the Fernández Espinosa SEP, BCR SEP and Chester’s SEP are poorly comparable. The same class of contamination was determined only for Cd, Fe, Ni and Zn, whereas the same bioavailability category was determined only for Cd, Pb and Zn. Generally, the ecological risk indices calculated from the extraction results by Fernández Espinosa SEP and BCR SEP showed better comparability than those calculated according to Chester’s SEP and Fernández Espinosa SEP, or using Chester’s SEP and BCR SEP.
- The total cancer inhalation risk estimated on the basis of both total concentrations of As, Cd, Co, Cr, Ni and Pb and their water-soluble fractions for residents of cities with low or moderate pollution appeared to be lower than the maximum acceptable limit of 1 × 10^−4^. However, the total non-cancerogenic inhalation risk was acceptable only when the risk was assessed based on water-soluble fractions of As, Cd, Co, Cr and Mn. The total non-carcinogenic inhalation risk for residents of highly polluted cities exceeded the acceptable risk level by 1–2 orders of magnitude.

## Figures and Tables

**Table 1:** Most widely used original procedures for chemical fractionation of PTEs in atmospheric particulate matter.

SEP	Fraction	Analytical Procedure	Reference
Fernández Espinosa procedure	Soluble and exchangeable	(1) Water, shaking, RT, 5 h	[40]
	Carbonates, oxides and reducible	(2) 0.25 M NH_2_OH∙HCl (pH 2), shaking, RT, 5 h	
	Bound to organic matter, oxidizable and sulphidic	(3) (a) 30% H_2_O_2_, shaking, 95 °C, until nearly dry, (b) 30% H_2_O_2_, shaking, 95 °C, until nearly dry, (c) 2.5 M AcONH_4_ (pH 3), shaking, RT, 1.5 h	
	Residual	(4) HNO_3_, HCl, HClO_4_, 95 °C, 5 h	
Original BCR procedure	Exchangeable, water- and acid-soluble	(1) 0.11 M AcOH, shaking, RT, 16 h	[82]
	Reducible	(2) 0.1 M NH_2_OH·HCl (pH 2, HNO_3_), shaking, RT, 16 h	
	Oxidizable	(3) (a) 8.8 M H_2_O_2_ (pH 2–3), shaking, RT, 1 h, then 85 °C, 1 h (b) 8.8 M H_2_O_2_ (pH 2–3), shaking, 85 °C, 1 h, (c) 1 M AcONH_4_ (pH 2, HNO_3_), shaking, RT, 16 h	
	Residual	(4) (a) Aqua regia, RT, 16 h, (b) reflux, 2 h (additional, recommended stage)	
Optimized BCR procedure	Exchangeable, water- and acid-soluble	(1) 0.11 M AcOH, shaking, RT, 16 h	[83]
	Reducible	(2) 0.5 M NH_2_OH·HCl/0.05 M HNO_3_, shaking, RT, 16 h	
	Oxidizable	(3) (a) 8.8 M H_2_O_2_ (pH 2–3), shaking, RT, 1 h, then 85 °C, 1 h (b) 8.8 M H_2_O_2_ (pH 2–3), shaking, 85 °C, 1 h, (c) 1 M AcONH_4_ (pH 2, HNO_3_), shaking, RT, 16 h	
	Residual	(4) (a) *Aqua regia*, RT, 16 h, (b) reflux, 2 h (*additional, recommended stage*)	
Chester’s procedure	Loosely held	(1) 1 M AcONH_4_ (pH 7), RT, agitation, 15 min	[75]
	Carbonate and oxide	(2) 1 M NH_2_OH∙HCl/25% AcOH, RT, agitation, 6 h	
	Refractory and organic	(3) HNO_3_, HF, 100 °C	
Tessier procedure	Exchangeable	(1) 1 M MgCl_2_ (pH 7.0) or 1 M AcONa (pH 8.2), shaking, RT, 1 h	[84]
	Bound to carbonates	(2) 1 M AcONa (pH 5, AcOH), shaking, RT, 5 h	
	Bound to Fe and Mn oxides	(3) 0.04 M NH_2_OH∙HCl/25% AcOH, shaking, 96 °C, 6 h or 0.3 M Na_2_S_2_O_4_/0.175 M Na-citrate/0.025 M H-citrate, shaking, 96 °C, 6 h	
	Bound to organic matter	(4) (a) 30% H_2_O_2_ (pH 2, HNO_3_), shaking, 85 °C, 2 h, (b) 30% H_2_O_2_ (pH 2, HNO_3_), shaking, 85 °C, 3 h, (c) 3.2 M AcONH4/20% HNO_3_, shaking, 30 min	
	Residual	(5) (a) HF, HClO_4_, heating until nearly dry, (b) HF, HClO_4_, heating until nearly dry, (c) 12 M HCl, H_2_O	
Obiols procedure	Soluble and exchangeable	(1) 1% NaCl, shaking, RT, 30 min	[85]
	Carbonates, and oxides	(2) 0.04 M NH_2_OH∙HCl/25% AcOH, shaking, 95 °C, 1 h	
	Bound to organic matter	(3) (a) 0.02 M HNO_3_, 30% H_2_O_2_, 85 °C, 1 h, shaking, (b) 30% H_2_O_2_, (c) 3.2 M AcONH_4_/20% HNO_3_, shaking, RT	
	Residual	(4) HNO_3_, HCl, H_2_O, 95 °C, 1.5 h	
Wenzel procedure	Non-specifically sorbed	(1) 0.05 M (NH_4_)_2_SO_4_, shaking, 20 °C, 4 h	[86]
	Specifically sorbed	(2) 0.05 M (NH_4_)H_2_PO_4_, shaking, 20 °C, 16 h	
	Amorphous and poorly crystalline hydrous oxides of Fe and Al	(3) 0.2 M NH_4_-oxalate buffer, pH 3.25, shaking, dark, 20 °C, 4 h	
	Well-crystalized hydrous oxides of Fe and Al	(4) 0.2 M NH_4_-oxalate buffer, 0.1 M ascorbic acid, pH 3.25, 96 °C, 30 min	
	Residual	(5) HNO_3_, H_2_O_2_, microwave-assisted	

**Table 2:** Descriptive statistics of literature data of operational speciation of As, Cd, Co, Cr, Cu, Fe, Mn, Ni, Pb and Zn occurring in urban air particulate matter by Fernández Espinosa procedure. The element fraction content is given as a percentage of its total content [%].

Element Fraction	Number of Data Sets	Mean	SD	Median	Range	Kurtosis	Skewness
F(1)-As	18	29.2	22.8	31.0	2.4–98.1	3.58	1.38
F(2)-As		26.7	18.2	34.2	0.2–67.1	−0.28	0.07
F(3)-As		19.2	18.4	16.0	0.0–76.5	4.62	1.96
F(4)-As		25.0	28.9	14.0	0.0–93.1	0.80	1.50
F(1)-Cd	22	42.5	19.1	42.3	5.0–78.5	−0.34	−0.17
F(2)-Cd		24.7	15.1	20.4	3.5–67.3	1.87	1.28
F(3)-Cd		18.8	16.8	14.5	3.8–73.3	4.30	1.94
F(4)-Cd		14.0	11.4	11.1	0.0–48.8	2.89	1.32
F(1)-Co	12	35.7	14.0	37.6	15.7–54.3	−1.59	−0.12
F(2)-Co		19.3	7.6	21.3	5.7–29.0	−0.81	−0.41
F(3)-Co		22.3	10.5	18.5	11.8–43.4	−0.47	0.83
F(4)-Co		22.6	11.5	19.4	6.6–49.5	1.61	1.09
F(1)-Cr	19	14.6	15.7	8.2	0.0–46.5	−0.80	0.82
F(2)-Cr		10.6	11.5	9.2	0.8–51.0	8.56	2.58
F(3)-Cr		28.8	19.1	23.8	0.0–70.5	0.06	0.51
F(4)-Cr		46.0	29.6	50.5	0.0–98.8	−0.86	0.24
F(1)-Cu	15	22.6	11.0	20.0	0.0–38.0	−0.41	−0.19
F(2)-Cu		22.9	13.9	21.0	2.4–61.0	3.44	1.52
F(3)-Cu		28.1	14.5	30.2	3.5–51.9	−0.61	−0.12
F(4)-Cu		26.5	23.1	18.0	2.4–94.0	4.64	2.02
F(1)-Fe	14	6.5	7.7	3.9	0.3–29.6	6.34	2.30
F(2)-Fe		15.7	6.3	15.3	6.2–32.7	3.52	1.39
F(3)-Fe		26.9	12.4	25.4	7.7–48.0	−0.84	0.37
F(4)-Fe		51.0	11.4	50.7	33.5–70.6	−0.69	0.27
F(1)-Mn	18	30.8	17.4	33.3	1.1–55.3	−0.78	−0.58
F(2)-Mn		24.0	12.4	24.9	6.0–49.4	−0.06	0.53
F(3)-Mn		27.1	14.0	27.0	5.0–73.4	7.11	1.95
F(4)-Mn		18.1	18.1	13.2	3.9–81.2	9.08	2.77
F(1)-Ni	18	23.3	15.2	23.4	1.9–64.2	1.79	0.91
F(2)-Ni		24.6	20.0	17.9	4.7–65.3	0.72	1.39
F(3)-Ni		26.7	16.2	30.1	0.0–54.0	−1.13	−0.25
F(4)-Ni		25.3	20.2	17.0	6.0–73.3	2.00	1.72
F(1)-Pb	23	20.1	15.7	21.4	0.0–55.0	−0.16	0.59
F(2)-Pb		39.0	17.4	41.0	5.0–73.9	−0.07	−0.14
F(3)-Pb		17.1	11.2	16.5	1.2–49.9	2.18	1.09
F(4)-Pb		23.7	24.6	14.0	1.3–76.5	0.33	1.26
F(1)-Zn	16	32.3	21.3	37.1	0.1–61.4	−1.46	−0.20
F(2)-Zn		24.0	12.0	27.1	0.7–45.2	−0.11	−0.52
F(3)-Zn		21.1	13.5	15.6	6.2–49.5	−0.23	0.98
F(4)-Zn		22.8	21.0	15.3	0.5–58.8	−1.17	0.68

**Table 3:** Descriptive statistics of literature data of operational speciation of As, Cd, Co, Cr, Cu, Fe, Mn, Ni, Pb and Zn occurring in urban air particulate matter by BCR procedure. The element fraction content is given as a percentage of its total content [%].

Element Fraction	Number of Data Sets	Mean	SD	Median	Range	Kurtosis	Skewness
F(1)-As	10	37.8	22.7	45.1	6.4–72.0	−0.94	−0.46
F(2)-As		24.8	13.7	27.3	2.9–44.2	−1.06	−0.20
F(3)-As		15.9	15.1	8.6	3.7–44.3	0.28	1.34
F(4)-As		21.5	22.1	14.0	6.7–78.4	5.48	2.30
F(1)-Cd	19	47.3	16.0	51.3	16.8–82.3	0.06	0.12
F(2)-Cd		14.2	11.2	11.3	0.0–38.0	−0.63	0.59
F(3)-Cd		21.4	17.0	17.4	0.0–59.0	−0.43	0.71
F(4)-Cd		16.9	11.3	16.5	1.0–44.4	0.80	0.75
F(1)-Co	11	15.9	6.5	15.8	6.6–32.2	4.16	1.54
F(2)-Co		13.3	6.4	10.9	5.5–22.6	−1.62	0.47
F(3)-Co		30.8	11.9	32.8	7.2–43.4	−0.13	−0.91
F(4)-Co		40.0	20.6	43.0	13.6–80.7	−0.31	0.56
F(1)-Cr	17	13.0	12.8	12.4	0.7–51.1	4.02	1.68
F(2)-Cr		21.3	17.2	17.3	0.0–62.0	0.57	1.03
F(3)-Cr		22.8	13.6	22.6	3.4–51.1	0.06	0.61
F(4)-Cr		42.8	24.5	35.6	8.4–95.0	−0.21	0.67
F(1)-Cu	20	31.6	19.9	29.5	2.2–76.7	−0.21	0.60
F(2)-Cu		16.5	6.9	15.2	2.0–29.3	−0.18	−0.07
F(3)-Cu		24.7	12.8	25.4	3.4–51.8	−0.13	0.44
F(4)-Cu		27.3	20.2	23.1	3.0–88.9	3.51	1.60
F(1)-Fe	8	9.8	6.4	8.0	1.3–18.3	−1.65	0.22
F(2)-Fe		20.1	13.8	14.9	7.0–42.6	−0.24	1.21
F(3)-Fe		22.3	21.2	16.8	3.2–65.0	1.47	1.44
F(4)-Fe		47.9	24.0	48.9	18.7–79.6	−1.90	0.00
F(1)-Mn	13	30.5	13.6	33.8	8.1–57.0	−0.13	−0.02
F(2)-Mn		18.5	20.5	9.6	4.0–70.3	3.40	2.09
F(3)-Mn		19.7	10.6	20.0	0.0–36.4	−0.54	−0.19
F(4)-Mn		31.3	16.1	31.0	13.5–64.5	0.36	0.99
F(1)-Ni	14	12.8	7.4	10.5	4.0–28.5	0.25	1.03
F(2)-Ni		17.4	7.5	17.7	2.5–27.4	−0.68	−0.38
F(3)-Ni		35.5	15.7	40.6	5.0–52.1	−0.25	−1.02
F(4)-Ni		34.3	23.5	24.3	14.9–84.3	1.14	1.50
F(1)-Pb	19	34.0	25.7	30.9	3.0–85.8	−0.89	0.53
F(2)-Pb		26.1	11.9	23.3	10.2–49.0	−0.71	0.54
F(3)-Pb		18.9	17.3	15.5	1.1–63.7	3.04	1.83
F(4)-Pb		20.9	23.6	9.9	0.0–77.1	0.95	1.33
F(1)-Zn	20	47.1	23.5	54.5	8.0–85.9	−1.05	−0.19
F(2)-Zn		15.8	9.4	13.9	3.8–34.6	−0.83	0.52
F(3)-Zn		18.3	18.5	13.4	1.5–75.0	4.81	2.19
F(4)-Zn		18.7	17.0	10.0	0.5–55.0	−0.79	0.78

**Table 4:** Descriptive statistics of literature data of operational speciation of As, Cd, Co, Cr, Cu, Fe, Mn, Ni, Pb and Zn occurring in urban air particulate matter by Chester’s procedure. The element fraction content is given as a percentage of its total content [%].

Element Fraction	Number of Data Sets	Mean	SD	Median	Range	Kurtosis	Skewness
F(1)-As	7	29.1	26.8	19.0	3.0–70.1	−0.93	0.97
F(2)-As		17.4	14.1	14.0	2.0–36.3	−1.55	0.44
F(3)-As		53.5	37.9	67.0	0.0–94.0	−1.62	−0.53
F(1)-Cd	13	61.8	29.1	61.4	5.5–100.0	−0.25	−0.63
F(2)-Cd		12.2	11.3	10.4	0.0–30.0	−1.42	0.42
F(3)-Cd		26.0	30.0	13.6	0.0–94.0	1.63	1.65
F(1)-Cr	11	26.4	18.8	27.0	1.5–54.4	−1.35	0.13
F(2)-Cr		31.5	29.0	26.6	1.2–94.1	1.38	1.47
F(3)-Cr		42.1	30.5	40.0	1.0–97.0	−0.37	0.48
F(1)-Cu	15	31.9	24.8	22.8	3.7–91.2	0.79	1.14
F(2)-Cu		28.2	15.5	26.0	5.4–63.0	0.25	0.62
F(3)-Cu		39.9	19.0	43.2	3.4–75.7	−0.35	−0.05
F(1)-Fe	12	6.1	5.5	3.5	0.0–18.0	0.19	0.98
F(2)-Fe		28.2	24.6	21.9	2.9–75.0	−0.06	1.00
F(3)-Fe		65.7	27.9	73.1	18.0–96.2	−1.01	−0.66
F(1)-Mn	13	31.0	22.5	27.1	5.0–75.1	−0.59	0.64
F(2)-Mn		16.2	10.4	19.3	2.0–34.2	−1.13	0.00
F(3)-Mn		52.9	24.6	45.4	14.5–90.0	−1.06	0.28
F(1)-Ni	10	25.0	13.0	27.5	0.7–48.0	0.79	−0.24
F(2)-Ni		23.4	13.1	23.4	1.1–42.0	−0.48	0.01
F(3)-Ni		51.6	23.5	56.7	19.0–98.0	0.48	0.39
F(1)-Pb	14	61.8	23.1	61.4	5.7–93.7	1.41	−0.96
F(2)-Pb		20.8	11.7	17.7	4.5–46.1	0.25	0.88
F(3)-Pb		17.4	17.8	9.2	1.7–65.5	3.20	1.71
F(1)-Zn	12	50.6	30.7	44.0	0.2–94.5	−1.14	−0.07
F(2)-Zn		16.6	11.3	15.3	0.0–36.2	−1.07	0.21
F(3)-Zn		32.8	27.5	27.2	2.4–91.4	0.24	1.02

**Table 5:** The mean concentrations and the percentage of the water-soluble fraction of the ten PTEs in the air in urban areas around the world.

Element	Air Quality Standards [ng/m^3^]	Commonly Encountered Pollution Level									High Pollution Level		
		Range [ng/m^3^]	Mean Conc. ± SEM [ng/m^3^]	Median [ng/m^3^]	Kurtosis	Skewness	Water-Soluble Fraction [%]	Median [%]	Kurtosis	Skewness	Range [ng/m^3^]	Mean Conc. ± SEM [ng/m^3^]	Water-Soluble Fraction [%]
As	6 ^(1)^	0–50	19 ± 3 (*n* = 32)	18	−1.3681	0.3544	46 (*n* = 32)	44	0.2564	0.2698	>50	1.5 × 10^3^ ± 1.4 × 10^3^ (*n* = 8)	21 (*n* = 8)
Cd	5 ^(1,2)^	0–10	3.3 ± 0.5 (*n* = 37)	2.1	−1.0456	0.6879	54 (*n* = 40)	60	−0.3866	−0.6045	>10	9.6 × 10^1^ ± 4.3 × 10^1^ (*n* = 14)	41 (*n* = 15)
Co		0–10	1.2 ± 0.5 (*n* = 20)	0.4	8.8310	2.9437	37 (*n* = 21)	30	1.7134	1.2002	>10	1.8 × 10^2^ ± 0.7 × 10^2^ (*n* = 7)	29 (*n* = 7)
Cr		0–50	13 ± 2 (*n* = 31)	9.3	0.6823	1.0271	18 (*n* = 34)	12	−0.2495	0.8251	>50	3.5 × 10^2^ ± 0.7 × 10^2^ (*n* = 12)	5.4 (*n* = 13)
Cu		0–100	44 ± 6 (*n* = 32)	38	−1.3064	0.3713	29 (*n* = 35)	30	1.8778	0.8686	>100	1.3 × 10^3^ ± 0.6 × 10^3^ (*n* = 19)	22 (*n* = 20)
Fe		0–1000	46 × 10^1^ ± 7 × 10^1^ (*n* = 18)	38 × 10	−1.3077	0.3265	8.9 (*n* = 18)	7.1	8.3319	2.5542	>1000	2.6 × 10^3^ ± 0.4 × 10^3^ (*n* = 23)	5.5 (*n* = 24)
Mn	150 ^(3)^	0–100	38 ± 6 (*n* = 32)	20	−1.3318	0.6144	38 (*n* = 34)	39	2.0410	0.4390	>100	2.2 × 10^2^ ± 0.3 × 10^2^ (*n* = 17)	36 (*n* = 17)
Ni	20 ^(1)^	0–100	16 ± 4 (*n* = 34)	8.3	4.1668	1.9957	28 (*n* = 32)	27	1.3585	1.1514	>100	3.6 × 10^2^ ± 1.5 × 10^2^ (*n* = 8)	11 (*n* = 9)
Pb	500 ^(2)^	0–500	13 × 10^1^ ± 2 × 10^1^ (*n* = 57)	8 × 10	0.3486	1.1766	21 (*n* = 60)	20	−0.2814	0.5933	>500	3.9 × 10^3^ ± 2.6 × 10^3^ (*n* = 6)	24 (*n* = 5)
Zn		0–1000	26 × 10^1^ ± 4 × 10^1^ (*n* = 46)	17 × 10	0.7063	1.1090	52 (*n* = 46)	52	−1.1357	−0.1086	>1000	3.4 × 10^4^ ± 3.2 × 10^4^ (*n* = 3)	31 (*n* = 4)

^(1)^ EU target value [89]; ^(2)^ EU limit value [90]; ^(3)^ WHO guideline value [91].

**Table 6:** Mean proportion of the non-resistant fraction of ten PTEs in urban particulate matter worldwide, and corresponding bioavailability indices and individual contamination factors.

Element	Fernández Espinosa SEP					BCR SEP					Chester’s SEP				
	F(1/2) [%]	BI		ICF		F(1/2) [%]	BI		ICF		F(1/2) [%]	BI		ICF	
		Value	Bio-Availability	Value	Class of Contamination		Value	Bio- Availability	Value	Class of Contamination		Value	Bio-Availability	Value	Class of Contamination
As	55.9	0.56	high	1.3	moderate	62.6	0.63	high	1.7	moderate	46.5	0.46	medium	0.87	low
Cd	67.2	0.67	high	2.0	moderate	61.5	0.62	high	1.6	moderate	74.0	0.74	high	2.8	moderate
Co	55.0	0.55	high	1.2	moderate	29.2	0.29	low	0.41	low	73.2	0.73	high	4.4	considerable
Cr	25.2	0.25	low	0.34	low	34.3	0.34	medium	0.52	low	57.9	0.58	high	1.4	moderate
Cu	45.5	0.46	medium	0.83	low	48.1	0.48	medium	0.92	low	60.1	0.60	high	1.5	moderate
Fe	22.2	0.22	low	0.28	low	29.9	0.30	low	0.43	low	34.3	0.34	medium	0.52	low
Mn	54.8	0.55	high	1.2	moderate	49.0	0.49	medium	0.96	low	47.2	0.47	medium	0.89	low
Ni	47.9	0.48	medium	0.92	low	30.2	0.30	low	0.43	low	48.4	0.48	medium	0.94	low
Pb	59.1	0.59	high	1.5	moderate	60.1	0.60	high	1.5	moderate	82.6	0.83	high	4.7	considerable
Zn	56.3	0.56	high	1.3	moderate	62.9	0.63	high	1.7	moderate	67.2	0.67	high	2.9	moderate

**Table 7:** Values of parameters used in the health risk assessment.

Parameter	Acronym	Unit	Values	Reference
Exposure duration	*ED*	year	24	[98]
Exposure time	*ET*	h/day	24	[98]
Exposure frequency	*EF*	day/year	350	[98]
Average exposure time	*AT_n_*			
	For carcinogens	hours	613 × 200	[98]
	For non-carcinogens	hours	210 × 240	[98]
Inhalation unit risk	*IUR*			
As		(μg/m^3^)^−1^	4.30 × 10^−3^	[99]
Cd		(μg/m^3^)^−1^	1.80 × 10^−3^	[99]
Pb		(μg/m^3^)^−1^	1.20 × 10^−5^	[99]
Co		(μg/m^3^)^−1^	9.00 × 10^−3^	[99]
Ni		(μg/m^3^)^−1^	2.40 × 10^−4^	[99]
Cr		(μg/m^3^)^−1^	1.20 × 10^−2^	[99]
Reference concentration	*RfC*			
As		mg/m^−3^	1.50 × 10^−5^	[99]
Cd		mg/m^−3^	1.00 × 10^−5^	[99]
Mn		mg/m^−3^	5.00 × 10^−5^	[99]
Cr		mg/m^−3^	1.00 × 10^−4^	[99]
Ni		mg/m^−3^	9.00 × 10^−5^	[99]
Co		mg/m^−3^	6.00 × 10^−6^	[99]

**Table 8:** Carcinogenic risk from metallic and metalloid elements via inhalation exposure to airborne particles.

Element	*IUR* [μg/m^3^]^−1^	Commonly Encountered Pollution Level				High Pollution Level				*CR*			
		*C_i_* [ng m^−3^]		*EC_i_* [ng m^−3^]		*C_i_* [ng m^−3^]		*EC_i_* [ng m^−3^]		Commonly Encountered Pollution Level		High Pollution Level	
		Mean Conc.	Water-Soluble Fraction	Mean Conc.	Water-Soluble Fraction	Mean Conc.	Water-Soluble Fraction	Mean Conc.	Water-Soluble Fraction	Mean Conc.	Water-Soluble Fraction	Mean Conc.	Water-Soluble Fraction
As	4.30 × 10^−3^	19	8.7	6.3	2.9	1.5 × 10^3^	3.2 × 10^2^	4.9 × 10^2^	1.0 × 10^2^	2.7 × 10^−5^	1.3 × 10^−5^	2.1 × 10^−3^	4.3 × 10^−4^
Cd	1.80 × 10^−3^	3.3	1.8	1.1	5.9 × 10^−1^	9.6 × 10^1^	3.9 × 10^1^	3.2 × 10^1^	1.3 × 10^1^	2.0 × 10^−6^	1.1 × 10^−6^	5.8 × 10^−5^	2.3 × 10^−5^
Co	9.00 × 10^−3^	1.2	4.4 × 10^−1^	4.0 × 10^−1^	1.5 × 10^−1^	1.8 × 10^2^	5.2 × 10^1^	5.9 × 10^1^	1.7 × 10^1^	3.6 × 10^−6^	1.4 × 10^−6^	5.3 × 10^−4^	1.5 × 10^−4^
Cr	1.20 × 10^−2^	13	2.3	4.3	7.6 × 10^−1^	3.5 × 10^2^	1.9 × 10^1^	1.1 × 10^2^	6.2	5.2 × 10^−5^	9.1 × 10^−6^	1.3 × 10^−3^	7.4 × 10^−5^
Ni	2.40 × 10^−4^	16	4.5	5.3	1.5	3.6 × 10^2^	3.9 × 10^1^	1.2 × 10^2^	1.3 × 10^1^	1.3 × 10^−6^	3.6 × 10^−7^	2.9 × 10^−5^	3.1 × 10^−6^
Pb	1.20 × 10^−5^	1.3 × 10^2^	27	43	8.9	3.9 × 10^3^	9.4 × 10^2^	1.3 × 10^3^	3.1 × 10^2^	5.2 × 10^−7^	1.1 × 10^−7^	1.6 × 10^−5^	3.7 × 10^−6^
*TCR*										8.6 × 10^−^^5^	2.5 × 10^−5^	4.0 × 10^−3^	6.8 × 10^−4^

**Table 9:** Non-carcinogenic risk from metallic and metalloid elements via inhalation exposure to airborne particles.

Element	*RfC* [mg/m^3^]	Commonly Encountered Pollution Level				High Pollution Level				*HQ*			
		*C_i_* [ng m^−3^]		*EC_i_* [ng m^−3^]		*C_i_* [ng m^−3^]		*EC_i_* [ng m^−3^]		Commonly Encountered Pollution Level		High Pollution Level	
		Mean Conc.	Water-Soluble Fraction	Mean Conc.	Water-Soluble Fraction	Mean Conc.	Water-Soluble Fraction	Mean Conc.	Water-Soluble Fraction	Mean Conc.	Water-Soluble Fraction	Mean Conc.	Water-Soluble Fraction
As	1.50 × 10^−^^5^	19	8.7	18	8.4	1.5 × 10^3^	3.2 × 10^2^	1.4 × 10^3^	3.0 × 10^2^	1.2	5.6 × 10^−1^	93	20
Cd	1.00 × 10^−5^	3.3	1.8	3.2	1.7	9.6 × 10^1^	3.9 × 10^1^	9.2 × 10^1^	3.7 × 10^1^	3.2 × 10^−1^	1.7 × 10^−1^	9.2	3.7
Co	6.00 × 10^−6^	1.2	4.4 × 10^−1^	1.2	4.2 × 10^−1^	1.8 × 10^2^	5.2 × 10^1^	1.7 × 10^2^	5.0 × 10^1^	2.0 × 10^−1^	7.0 × 10^−2^	28	8.3
Cr	1.00 × 10^−4^	13	2.3	12	2.2	3.5 × 10^2^	1.9 × 10^1^	3.4 × 10^2^	1.8 × 10^1^	1.2 × 10^−^^1^	2.2 × 10^−2^	3.4	1.8 × 10^−1^
Ni	9.00 × 10^−5^	16	4.5	15	4.3	3.6 × 10^2^	4.0 × 10^1^	3.5 × 10^2^	3.8 × 10^1^	1.7 × 10^−1^	4.7 × 10^−2^	3.9	4.2 × 10^−1^
Mn	5.00 × 10^−5^	38	14	36	13	2.2 × 10^2^	7.9 × 10^1^	2.1 × 10^2^	7.6 × 10^1^	7.2 × 10^−^^1^	2.6 × 10^−1^	4.2	1.5
*HI*										2.7	1.1	1.4 × 10^2^	34

## Data Availability

Data is contained within the article and supplementary material.

## Supplementary Materials

The following supporting information can be downloaded at: https://www.mdpi.com/article/10.3390/toxics10030124/s1, Figure S1: Operational speciation of As in urban atmospheric particulate matter based on literature data; Figure S2: Operational speciation of Cd in urban atmospheric particulate matter based on literature data; Figure S3: Operational speciation of Co in urban atmospheric particulate matter based on literature data; Figure S4: Operational speciation of Cr in urban atmospheric particulate matter based on literature data; Figure S5: Operational speciation of Cu in urban atmospheric particulate matter based on literature data; Figure S6: Operational speciation of Fe in urban atmospheric particulate matter based on literature data; Figure S7: Operational speciation of Mn in urban atmospheric particulate matter based on literature data; Figure S8: Operational speciation of Ni in urban atmospheric particulate matter based on literature data; Figure S9: Operational speciation of Pb in urban atmospheric particulate matter based on literature data; Figure S10: Operational speciation of Zn in urban atmospheric particulate matter based on literature data.
