# Supplementary material for: Chemical Fractionation in Environmental Studies of Potentially Toxic Particulate-Bound Elements in Urban Air: A Critical Review
Source: Toxics. 2022 Mar 4;10(3):124. doi: 10.3390/toxics10030124 (PMC8948661; doi:10.3390/toxics10030124)
Supplement: Supplementary file 1 [file toxics-10-00124-s001.zip › toxics-1582868-supplementary.pdf]

toxics

MDPI

# Supplementary Materials: Chemical Fractionation in Environmental Studies of Potentially Toxic Particulate-Bound Elements in Urban Air: A Critical Review

**Ryszard Świetlik * and Marzena Trojanowska**

**Fernandez-Espinoza procedure**

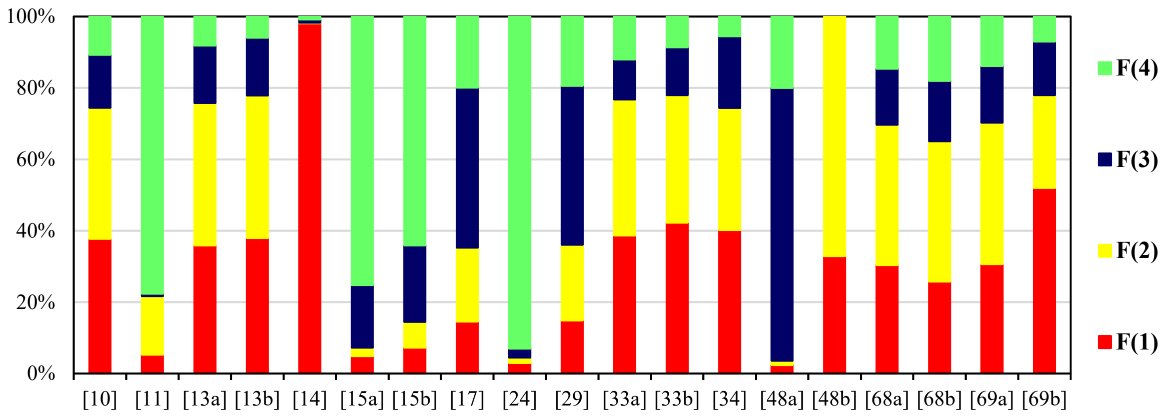


**BCR procedures**

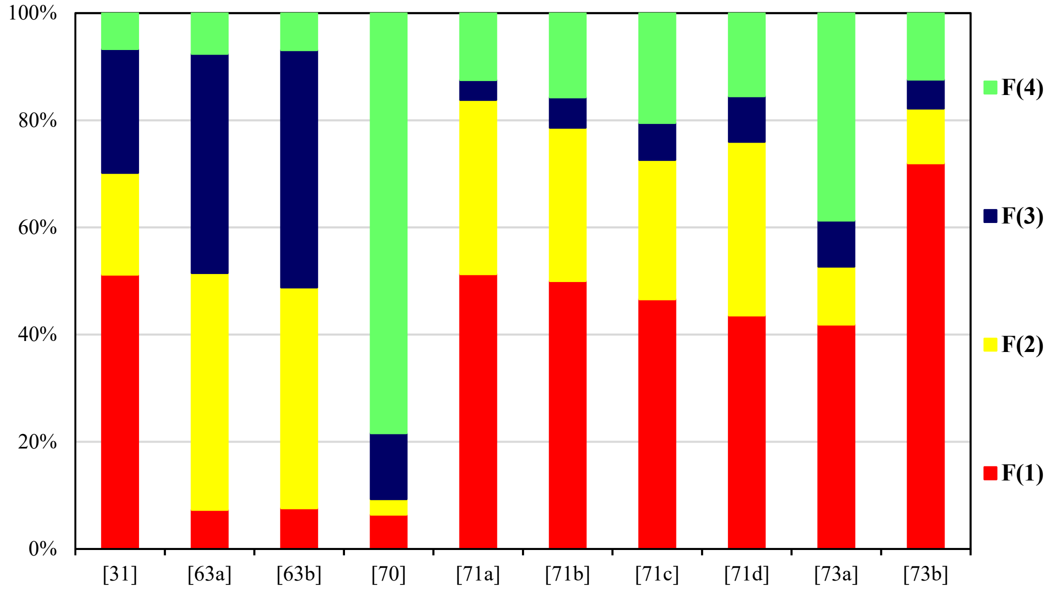

**Chester's procedure**

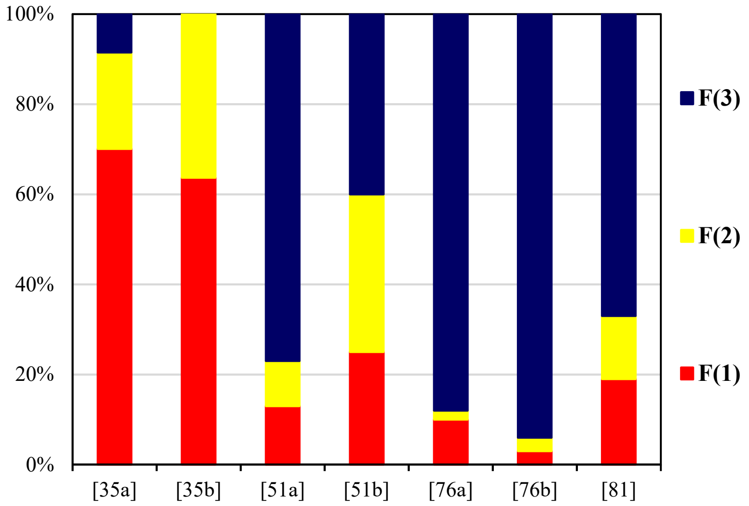


**Figure S1**. Operational speciation of As in urban atmospheric particulate matter based on literature data. The indexes *a, b, c* and d represent different data sets in the same article.

**Fernandez-Espinoza procedure**

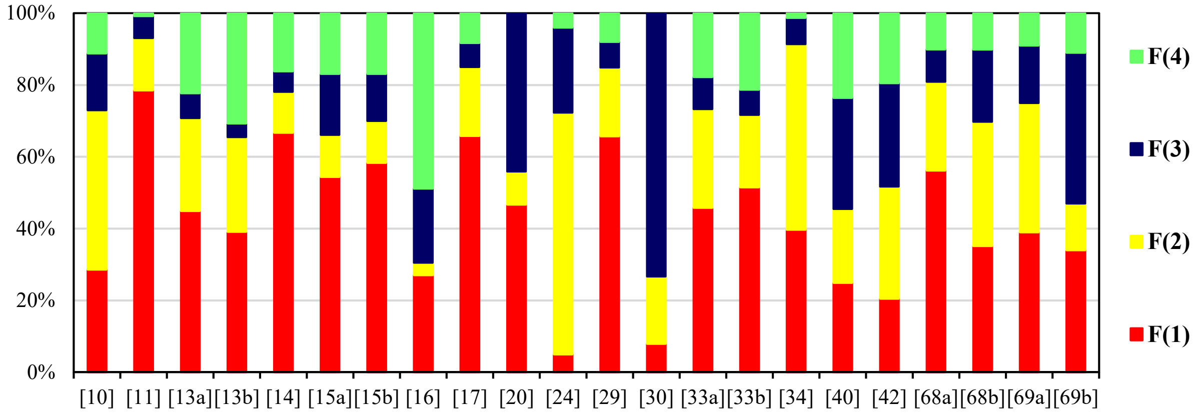


**BCR procedures**

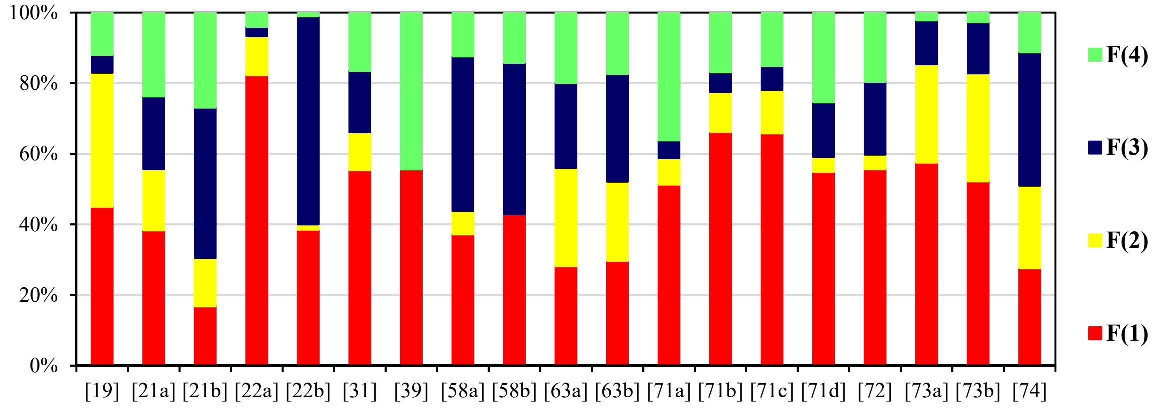

**Chester's procedure**

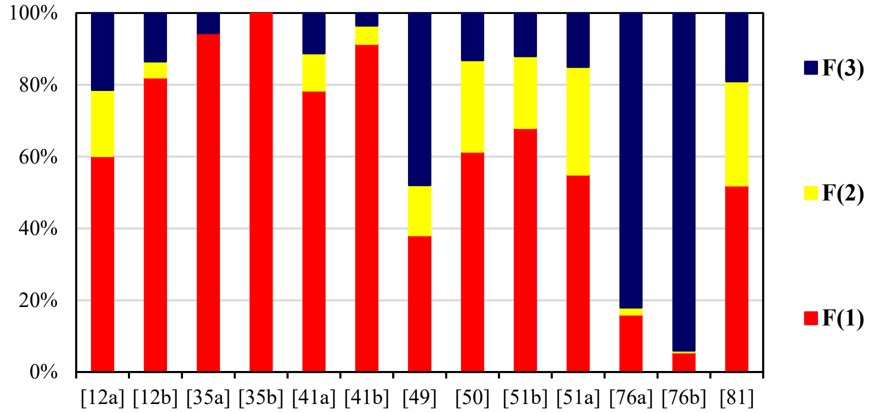


**Figure S2**. Operational speciation of Cd in urban atmospheric particulate matter based on literature data. The indexes *a, b, c* and d represent different data sets in the same article.

**Fernandez-Espinoza procedure**

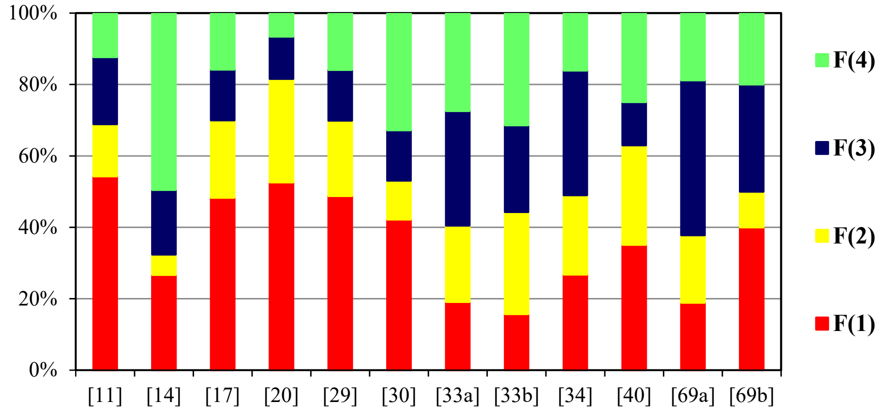


**BCR procedures**

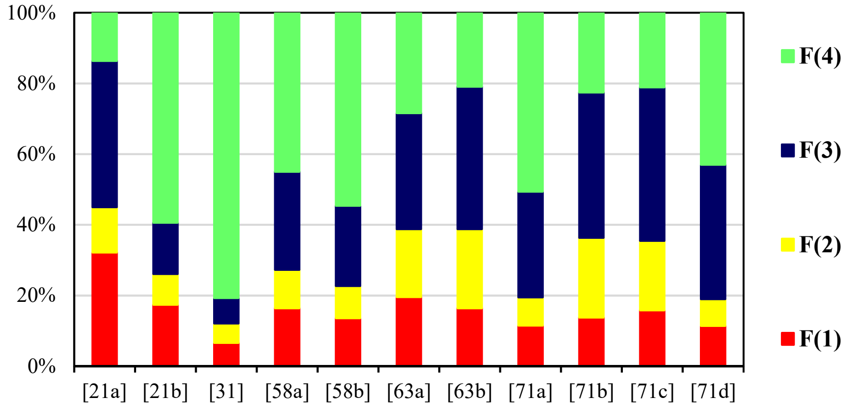

**Chester's procedure**

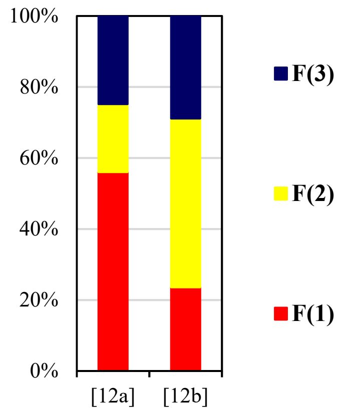


**Figure S3**. Operational speciation of Co in urban atmospheric particulate matter based on literature data. The indexes *a, b, c* and d represent different data sets in the same article.

**Fernandez-Espinoza procedure**

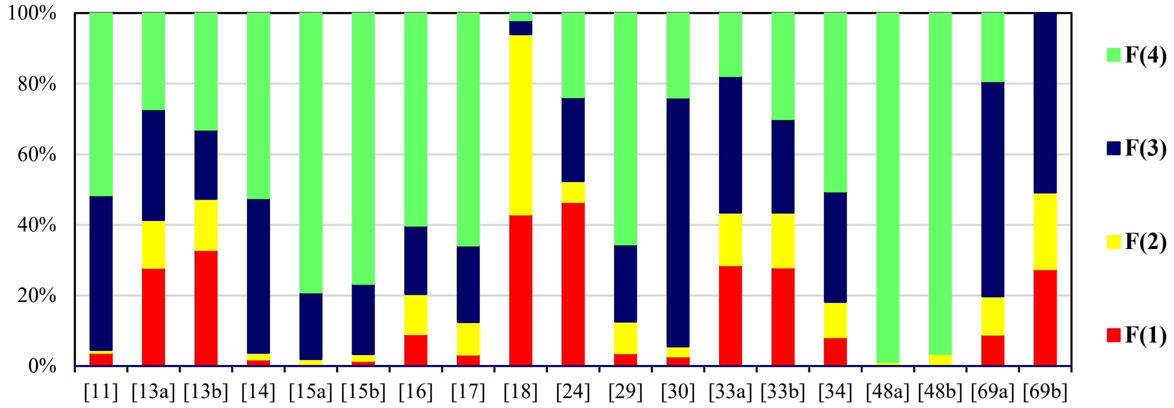


**BCR procedures**

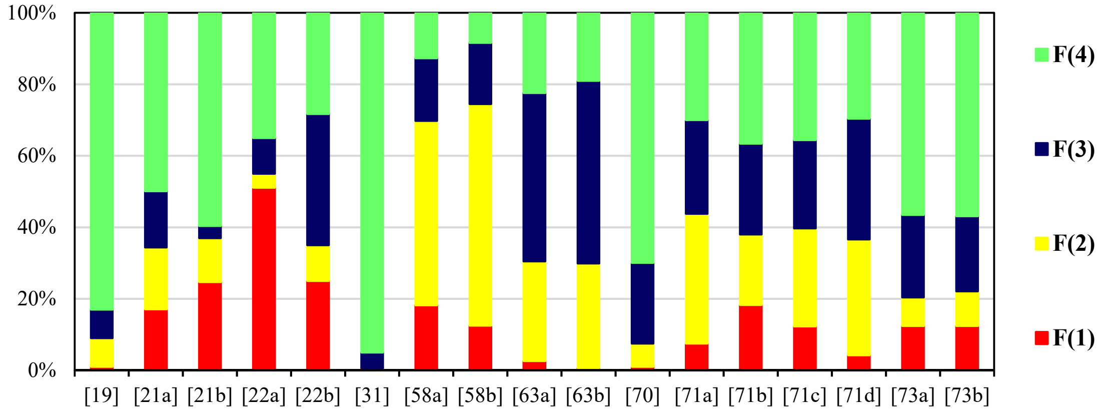

**Chester's procedure**

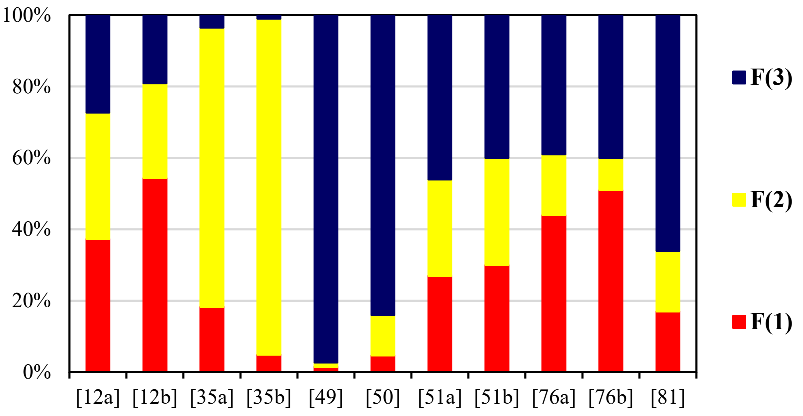


**Figure S4**. Operational speciation of Cr in urban atmospheric particulate matter based on literature data. The indexes *a, b, c* and d represent different data sets in the same article.

**Fernandez-Espinoza procedure**

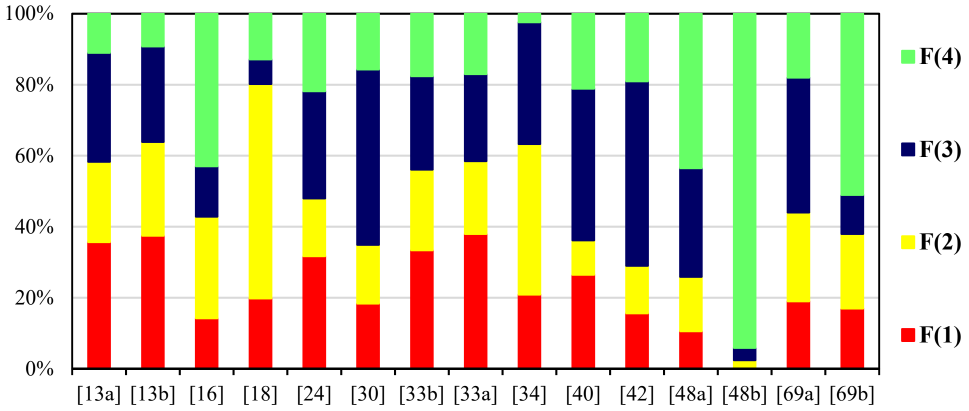


**BCR procedures**

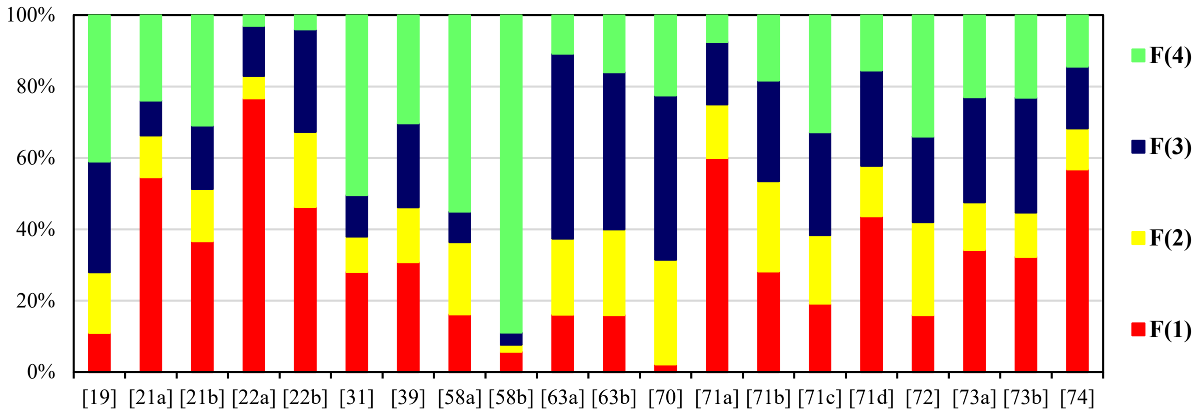

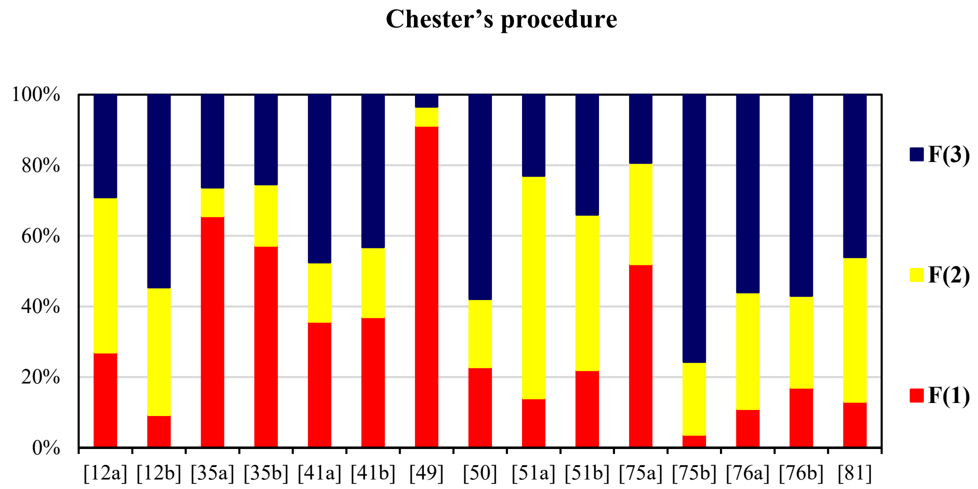


**Figure S5**. Operational speciation of Cu in urban atmospheric particulate matter based on literature data. The indexes *a, b, c* and d represent different data sets in the same article.

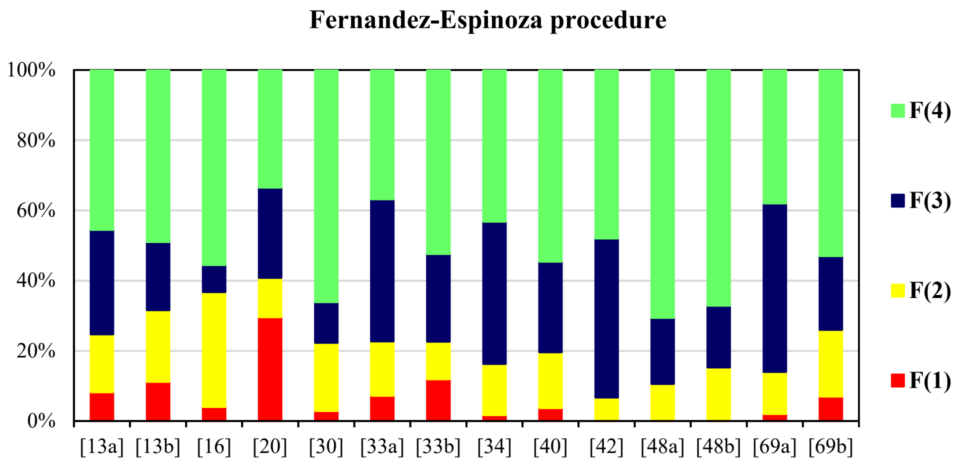


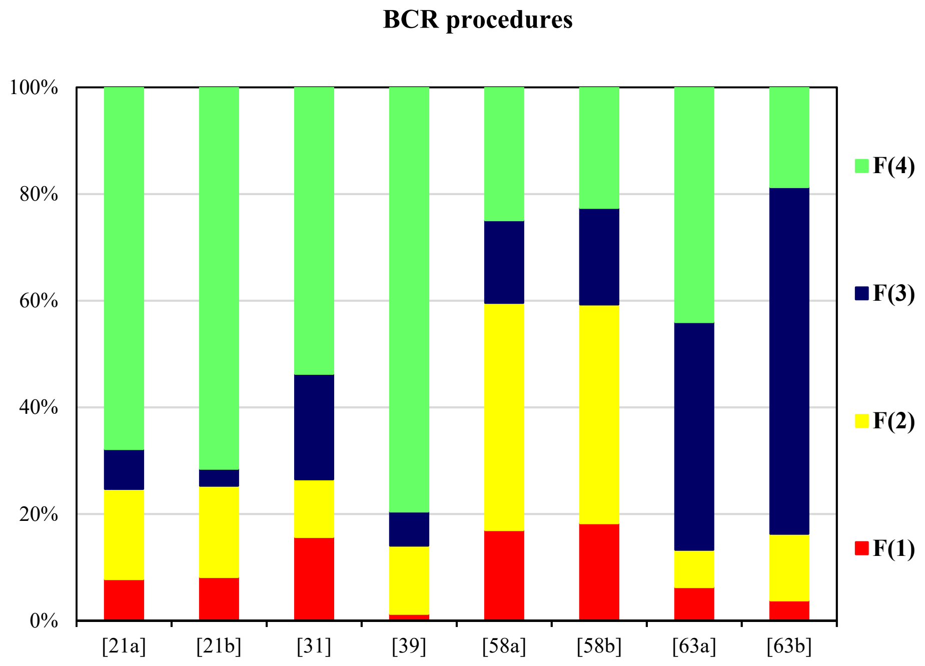

**Chester's procedure**

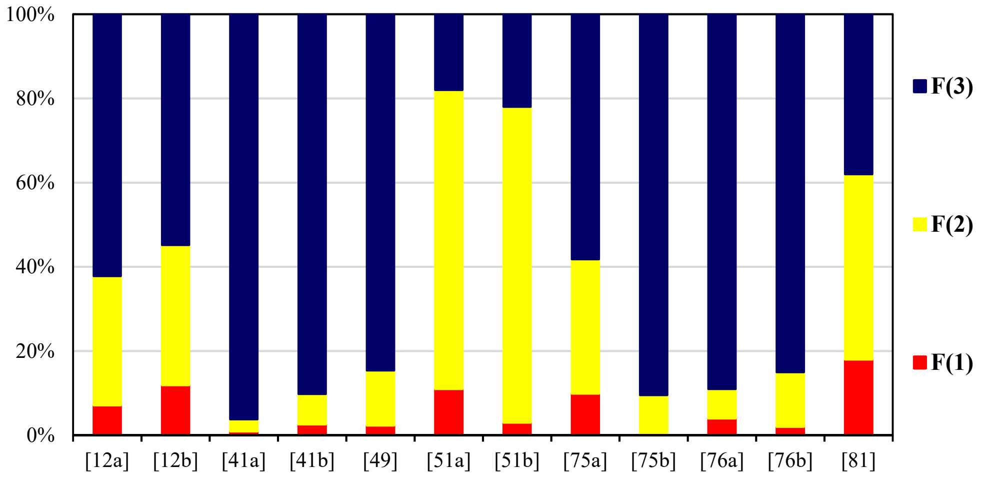


**Figure S6**. Operational speciation of Fe in urban atmospheric particulate matter based on literature data. The indexes *a, b, c* and d represent different data sets in the same article.

**Fernandez-Espinoza procedure**

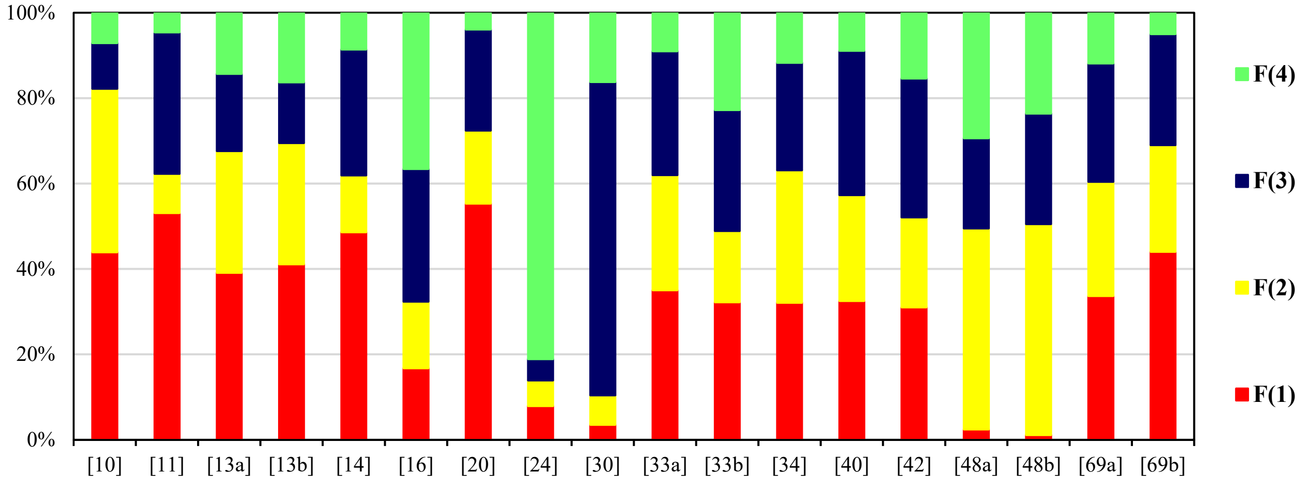


**BCR procedures**

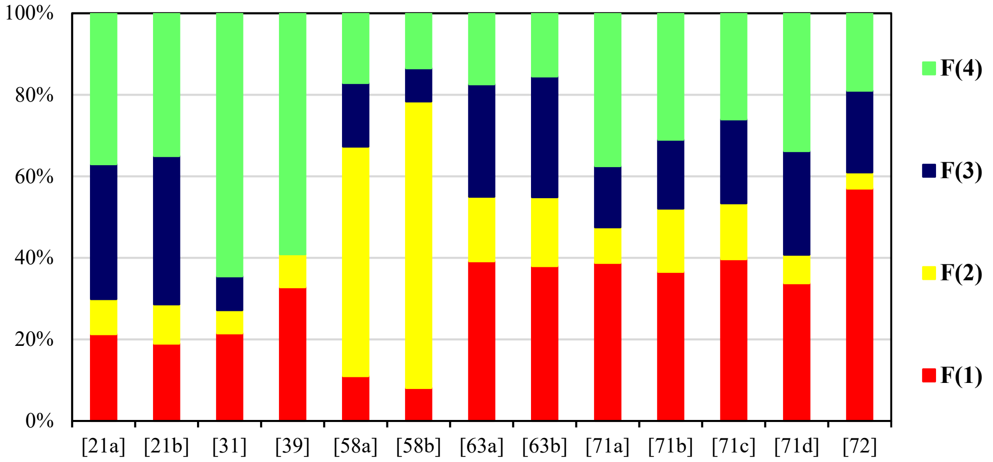


**Chester's procedure**

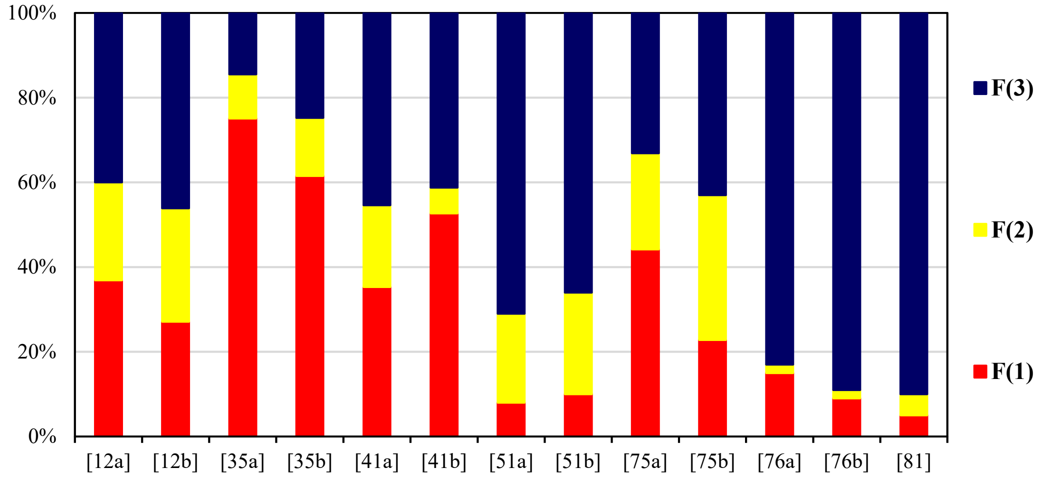


**Figure S7**. Operational speciation of Mn in urban atmospheric particulate matter based on literature data. The indexes *a, b, c* and d represent different data sets in the same article.

**Fernandez-Espinoza procedure**

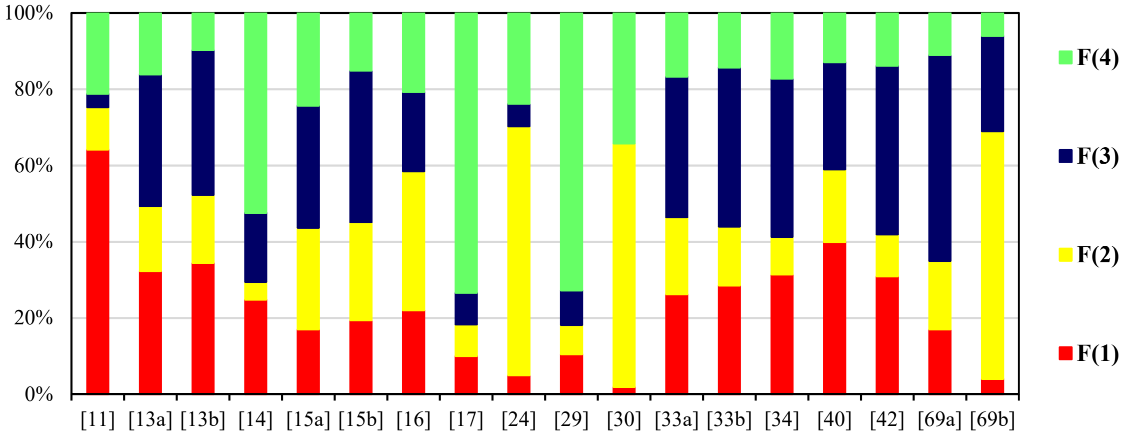

**BCR procedures**

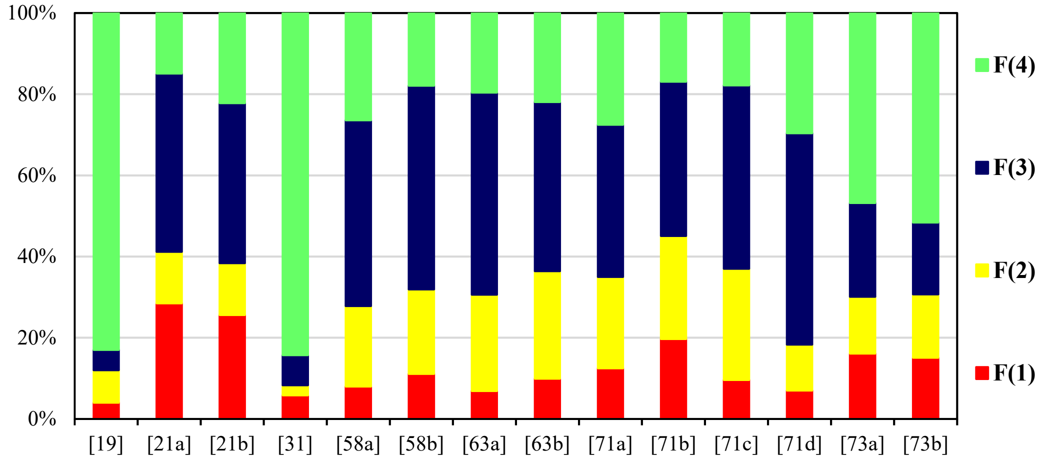


**Chester's procedure**

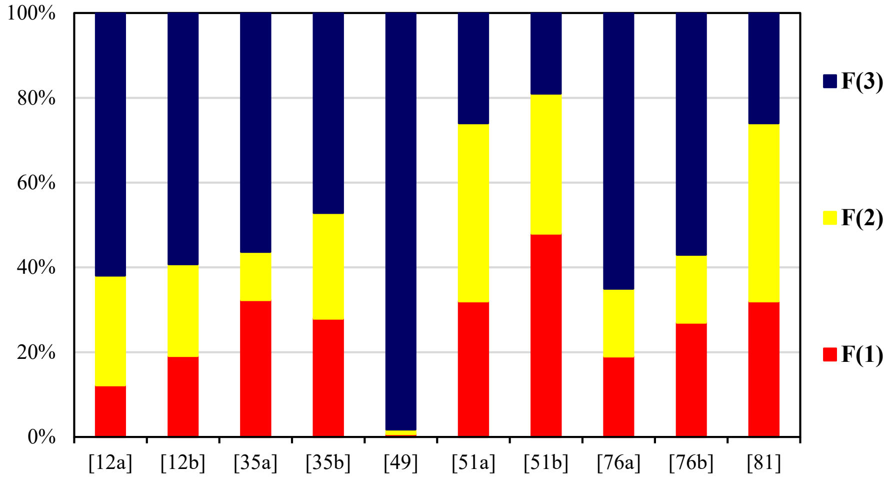


**Figure S8**. Operational speciation of Ni in urban atmospheric particulate matter based on literature data. The indexes *a, b, c* and d represent different data sets in the same article.

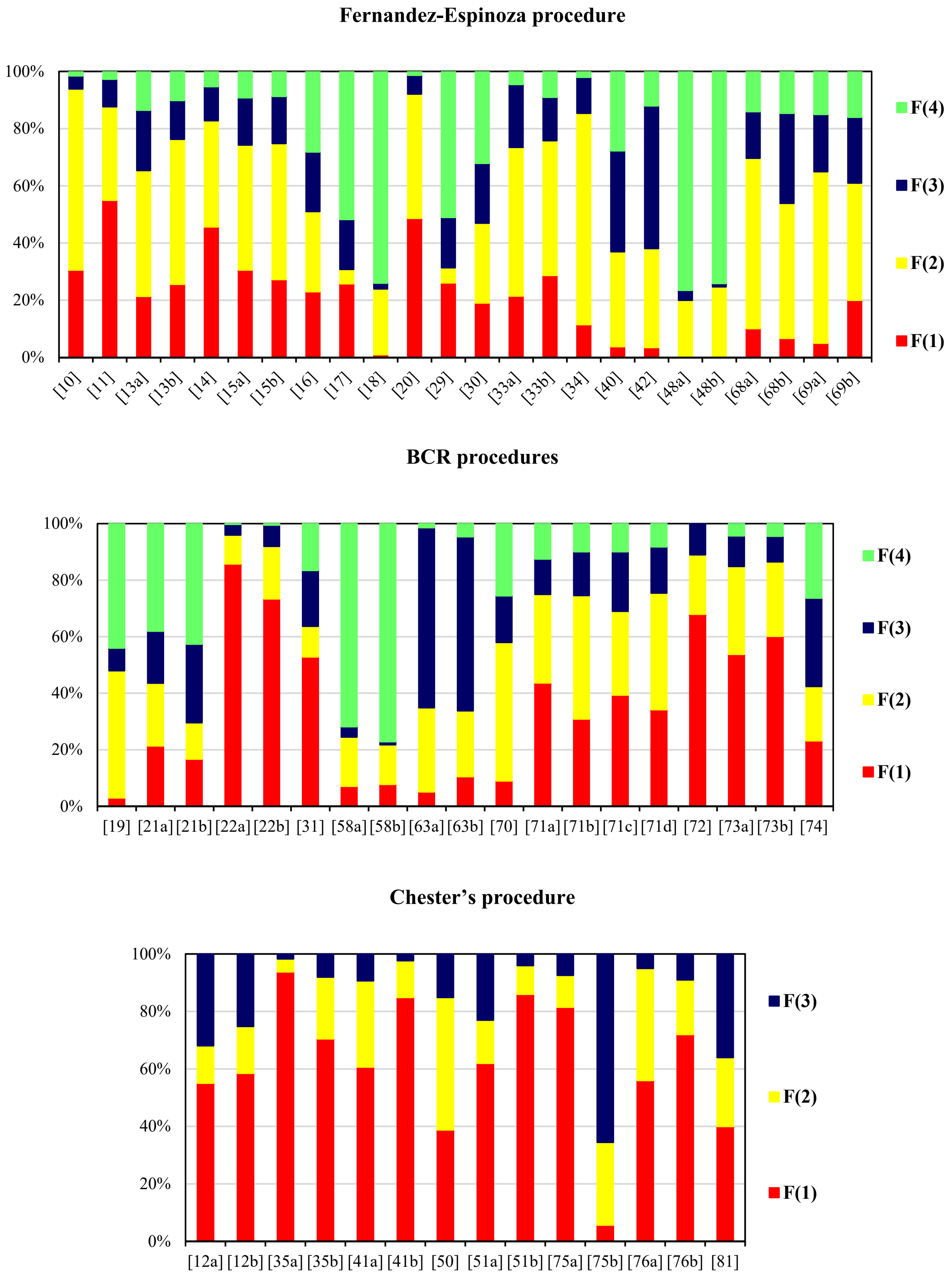


**Figure S9**. Operational speciation of Pb in urban atmospheric particulate matter based on literature data. The indexes *a, b, c* and d represent different data sets in the same article.

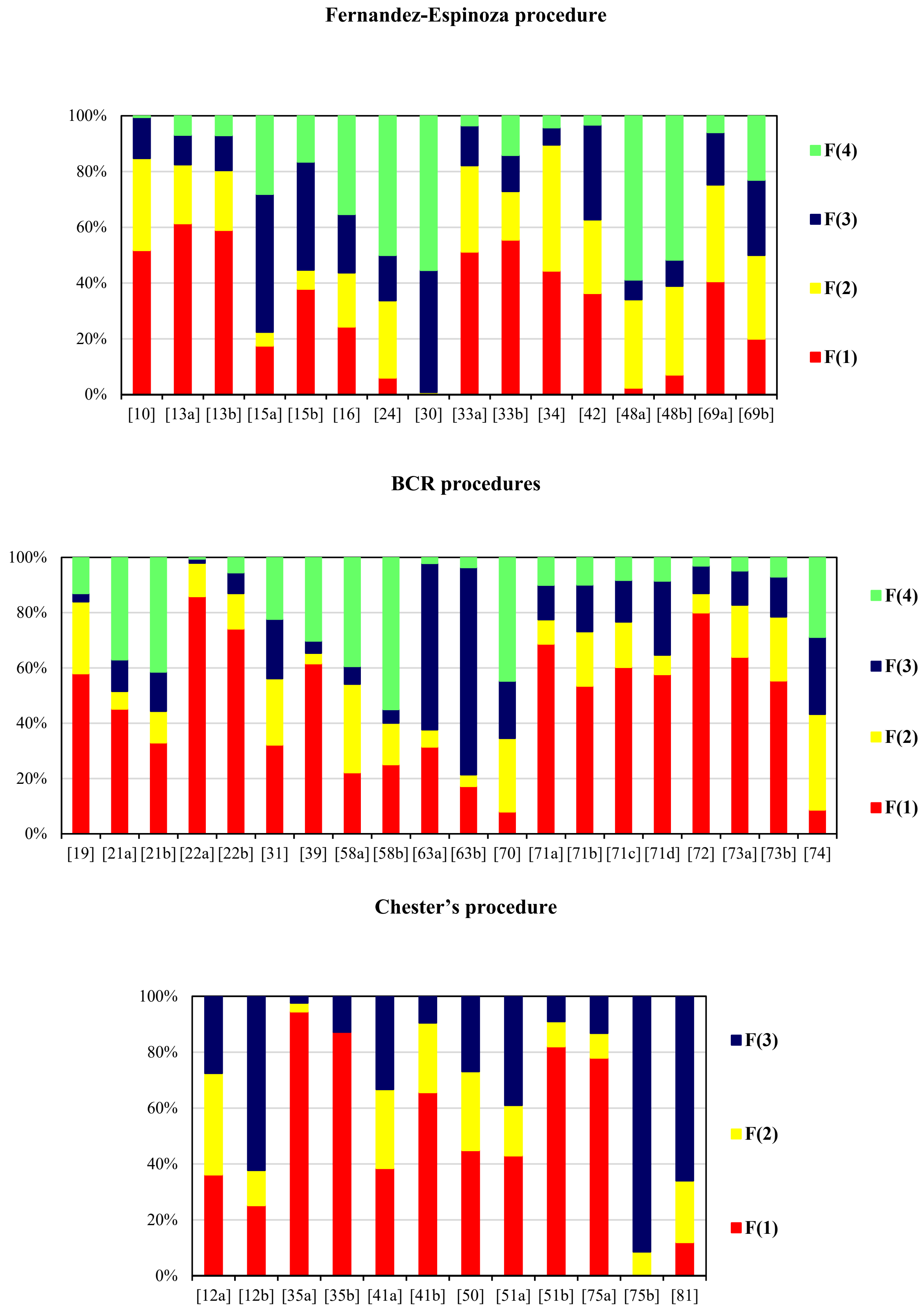


**Figure S10**. Operational speciation of Zn in urban atmospheric particulate matter based on literature data. The indexes *a, b, c* and d represent different data sets in the same article.
